# Impact of individual background on the unmet needs of cancer survivors and caregivers – a mixed-methods analysis

**DOI:** 10.1186/s12885-020-06732-5

**Published:** 2020-03-30

**Authors:** Kaname Watanabe, Kayoko Katayama, Takashi Yoshioka, Hiroto Narimatsu

**Affiliations:** 1 Department of Clinical Oncology, Yamagata University, Faculty of Medicine, Yamagata, Japan; 2 Cancer Prevention and Cancer Control Division, Kanagawa Cancer Center, Research Institute, 2-3-2 Nakao, Asahi-ku, Yokohama, Kanagawa 241-8515 Japan; 3 Graduate School of Health of Innovation, Kanagawa University of Human Services, Kawasaki, Kanagawa Japan

**Keywords:** Cancer survivors, Caregivers, Multivariate analysis, Qualitative research, Telephone

## Abstract

**Background:**

Cancer survivors and their caregivers may have various unmet needs that are medically difficult to solve. Previous studies have suggested the relations between individuals’ backgrounds and their unmet needs. We conducted a large-scale analysis to clarify the influence of individuals’ backgrounds, primarily cancer type, on specific types of unmet needs.

**Methods:**

Using a mixed-methods approach, we analyzed records of first-time callers to a cancer-focused telephone consultation service that was provided by the Kanagawa Cancer Clinical Research Information Organization from October 2006 to May 2014. The qualitative approach concerned extracting unmet needs mentioned in each consultation and classifying them into themes of specific needs, while the quantitative approach comprised multi-variated analysis of the relationships between the frequency by which the needs in each theme arose and the associated callers’ backgrounds.

**Results:**

A total of 1938 consultation cases were analyzed. In the qualitative analysis, the needs were classified into 16 themes. The mean number of unmet needs for each caller was 1.58 (standard deviation = 0.86). In the multi-variated analysis, caregivers for colorectal cancer survivors had a lower frequency of “emotional/mental health” needs (OR: 0.31, 95%CI: 0.11–0.88, *p* = 0.028) than did caregivers for breast-cancer survivors. Nevertheless, this was the only significant difference in needs frequency among callers (including survivors and their caregivers) with specific cancer types. Meanwhile, there significant difference in the frequency of occurrence of each unmet need theme was found among items concerning other background elements. Among survivors, sex was related to the frequency of needs among “physical” and “resources” themes, and “emotions/mental health”; their age group with “employment”; treatment course with “physical” and “resources” themes and “cure”; residence with “physical” themes; presence of symptom with “physical,” “education/information,” “resources,” “emotions/mental health,” and “cure” themes.

**Conclusions:**

This large-scale study suggests that cancer type is not a significant factor for specific unmet needs and that individuals’ backgrounds and presence of symptoms play a more important role. Through this study, it was found that instruments to predict people’s needs and a system to provide individualized cancer care across cancer types should be developed in the future.

## Background

Cancer survivors experience various difficulties in their daily lives, including physical, mental, social, and economic issues [1–4]. However, caregivers of cancer survivors (including family members, friends, and other people concerned) also encounter difficulties, and studies have shown that they can experience even greater anxiety than the survivors do [5–9]. Further, survivors of cancer, even when they are considered to be cured, often continue to experience difficulties associated with the disease, such as sequelae of treatment or psychosocial problems such as cancer stigma and changes in relationships with their acquaintances, which are medically difficult to solve [10–13]. Additionally, survivors and caregivers occasionally encounter difficulties in receiving adequate consultation time from medical providers, mainly due to time constraints, a desire not to trouble medical professionals, or communication problems. Thus, survivors and their caregivers have a range of unmet needs [14, 15].

Some studies have suggested that individuals’ backgrounds (age, sex, race, marital status, cancer type, treatment progress, treatment situation, etc.) are related to the specific types of unmet needs they experience [12, 16, 17]. This would be an important development for cancer care. If individuals’ backgrounds can be proven to predict specific types of unmet needs, we could determine the needs of survivors and caregivers in advance, and consequently provide adequate medical, psychosocial, and economic support. Such a predictive ability would also encourage survivors and caregivers to report their unmet needs and allow us to improve the efficiency of the care provided and make decisions based on survivors’/caregivers’ individual preferences. A few studies suggested a relationship between cancer type and specific unmet needs [16, 18–20], while the present study focused on the relationship. In clinical practice, cancers are conventionally dealt with by each category for the affected organ. Understanding how unmet needs vary by the type of cancer would make it possible to address those needs immediately and on an individual-basis, even under the current caregiving system.

Telephone consultation services are a promising means of identifying unmet needs. Such services allow medical professionals to respond to unmet needs that cannot be addressed through daily clinical practice [21–24]; for instance, these services allow survivors and caregivers to contact medical professionals even if they are geographically distant, are physically restricted, or do not wish to engage in face-to-face counseling [22, 25, 26]. Given their high utility, such services are widely used worldwide. Telephone consultations operate under a framework different from that of ordinary medical consultations, as the services are provided by professional consultation staff in locations remote from those of the survivors/caregivers, who can be contacted at any time.

Considering this, it is clear that records of telephone consultations are worth investigating because they potentially contain rich information regarding survivors’ and caregivers’ unmet medical needs that would otherwise be unattainable [6, 18, 27, 28]. There were some studies aiming to identify their medical needs from telephone counseling services [26, 29–32]; nevertheless, to our knowledge, no study was conducted to reveal the relationship between their unmet medical needs and individual background.

In this study, we qualitatively and quantitatively investigate the records of a cancer-focused telephone consultation service that was based in the Kanagawa Cancer Center Research Institute (KCCRI) in Japan. It was possible to conduct large-scale analysis of these data because a large amount of telephone consultation data was accumulated by the service, which allowed us to clarify the influence of individuals’ backgrounds on specific types of unmet needs.

## Methods

### Definitions

Some articles suggested various definitions of cancer survivors [12, 33]. In this study, we defined cancer survivors as people who were diagnosed with cancer and were still living, not including family members, friends, and caregivers. Caregivers were defined as people who have taken care of a cancer survivor, including family members, friends, and other people concerned.

### Telephone consultation service

In 2006, the Kanagawa Cancer Clinical Research Information Organization (KCCRIO), based in Yokohama, Japan, initiated a telephone counseling resource for cancer survivors and their caregivers. The KCCRIO was founded by public and private enterprises in October 2006 and remained in operation until May 2014, when the service was concluded on being integrated into similar telephone counseling service that newly launched. Although this telephone counseling service was based in Kanagawa and, therefore, it is likely that it was used primarily by Kanagawa residents, the service was also widely utilized by cancer survivors and caregivers located outside Kanagawa, both within Japan and overseas.

At KCCRIO, which was located in the KCCRI, three nurses with high levels of clinical experience worked in shifts to provide consultation and support via telephone, and the callers could consult a physician four days a week if necessary. The callers remained anonymous (i.e., they did not provide identifying information) and the consultation was provided free of charge (although standard telephone fees applied). Prior to the consultation, nurses obtained consent verbally to record (in written form) the content of the counseling for research purposes, and in some cases, survivors/caregivers provided an evaluation of the quality of the counseling they had received at the end of the call. While providing telephone counseling, nurses tallied survey items on a specialized form and recorded the content of the counseling. The forms recorded age (both that of the survivor in question and the caller, if applicable), sex (both that of the survivor in question and the caller, if applicable), the caller’s relationship with the survivor (if applicable), the time of the consultation, the number of previous consultations the caller had received, the survivor’s cancer type, the site of the survivor’s cancer, treatment progress, presence of symptoms, survivor’s (and caller’s, if applicable) area of residence, the time it would take the survivor to travel to a medical institute, and how the caller learned of this consultation service. The main topics of the counseling were recorded in more detail by the nurse after the counseling had finished. Microsoft Excel was used to store the data, with 13,962 consultations being provided by the service between October 2006 and May 2014. For the qualitative and quantitative analysis, we randomly extracted 3000 of the 13,962 consultations using a random number table generated by Microsoft Excel.

For the present study, to analyze these data, we chose to adopt a mixed-methods approach, combining qualitative analysis (involving classifying consultation content into specific themes regarding needs) with quantitative analysis (comprising statistical analysis of the correlation between needs and callers’ specific backgrounds).

Informed consent was obtained from all participant verbally since the subject was anonymous telephone counseling service. This study and the procedure for verbal consent were approved by the institutional review board of KCC (H27 epidemiology-12).

### Qualitative analysis

We identified individuals’ needs from the consultation records for each case and, by referring to the full transcript of each consultation, classified all needs into 16 need-related themes (physical, financial, education/information, personal control, system of care, resources, emotional/mental health, social support, communications, provider relationship, cure, body image, survivor identity, employment, and existential) to clarify the unmet medical needs of cancer survivors, which was proposed by Burg et al.; it was significant as a case of qualitative classification [12]. We calculated the total number of needs mentioned in each call, which was defined as the number of themes detected in the record for each call/case, in addition to calculating the frequency by which each theme appeared across all records. If we experienced difficulty classifying a need into an existing theme, or if there was no relevant theme, we decided to create new themes or redefine existing themes. For such difficult-to-classify needs, we held regular discussions on the best means of proceeding until a consensus was reached. We initially performed a pilot study in which we analyzed 1000 cases, and then, after discussions regarding the method, we added a further 2000 cases. All qualitative analyses were conducted by two authors (KW and HN).

### Quantitative analysis part

Following the qualitative analysis, we conducted quantitative analysis. Using a statistical approach, we examined the relationships among the mean values; frequency of occurrence of the needs; and the survivors’/caregivers’ demographic, psychosocial, and medical information.

Regarding the number of needs, we performed a Mann-Whitney U test and Kruskal-Wallis test to determine the differences in the mean number of unmet needs associated with each caller’s characteristics. We performed multivariable logistic regression analysis to determine the relationship between themes with a frequency greater than 1% and each characteristic. We primarily found that cancer type affected specific unmet needs. As a secondary analysis, and conducted a similar analysis of age, sex, relationship with survivor, and treatment course. At this point, to determine the differences in the unmet needs between survivors and caregivers, which were previously reported, and to prevent an influence of confounding factors, we divided eligible cases into survivors and other callers (mainly caregivers) for analysis, and focused on the survivors.

Statistical analyses were performed using EZR on R commander, version 2.13.0. *P* values less than 0.05 were considered to indicate statistical significance [34].

## Results

A total of 13,962 counseling cases were recorded by KCCRIO between October 2006 and May 2014; of these, 10,896 were first-consultation cases. We randomly sampled 3000 of the former cases. Given the aim of clarifying the distribution of unmet needs during telephone consultations, repeat callers’ consultations were considered inappropriate due to duplication of content. Therefore, after excluding 619 cases that were second (or higher) consultations and 443 cases whose context could not be interpreted due to lack of detailed information, we analyzed the remaining 1938 first-consultation cases. Table 1 shows the characteristics of the 1938 cases analyzed, as well as basic details regarding the 10,896 first-consultation cases. No items showed a significant difference (*p* < 0.05).

**Table 1:** Characteristics of the analysis subset in comparison to those of the full survey sample

	Analysis Subset	Total Surveyed
	(*n* = 1938)	(*n* = 11,044)
Caller’s sex, *n* (%)		
Male	637 (32.9)	3470 (31.5)
Female	1300 (67.1)	7542 (68.5)
Survivor’s sex, *n* (%)		
Male	719 (37.1)	4050 (42.0)
Female	995 (51.3)	5597 (58.0)
Caller’s age groups, *n* (%)		
< 40 y	282 (14.6)	1529 (14.5)
40–59 y	756 (39.0)	4294 (40.8)
≥ 60 y	830 (42.8)	4702 (44.6)
Survivor’s age groups, *n* (%)		
< 40 y	140 (7.2)	854 (9.8)
40–59 y	479 (24.7)	2673 (30.5)
60–69 y	459 (23.7)	2505 (28.6)
≥ 70 y	473 (24.4)	2718 (31.1)
Relationship with survivor, *n* (%)		
Survivor	1054 (54.4)	5867 (53.4)
Spouse	278 (14.3)	1771 (16.1)
Child	361 (18.6)	1961 (17.8)
Parent	54 (2.8)	337 (3.1)
Sibling	64 (3.3)	344 (3.1)
Other	124 (6.4)	708 (6.4)
Treatment course, *n* (%)		
Never diagnosed with cancer	492 (25.4)	2721 (28.8)
Ongoing	694 (35.8)	3937 (41.6)
Completed	468 (29.6)	2804 (29.6)
Cancer type, *n* (%)		
Breast	275(14.2)	1531 (14.1)
Lung	189 (9.8)	1061 (9.7)
Colorectal	181 (9.3)	1072 (9.8)
Stomach	156 (8.0)	918 (8.4)
Other	669 (34.5)	3705 (34.0)
Multi primary	59 (3.0)	345 (3.2)
Never diagnosed with cancer	409 (21.1)	2258 (20.7)
Residence, *n* (%)		
A city designated by ordinance	1162 (60.0)	6519 (61.3)
Within Kanagawa Prefecture	540 (27.9)	3173 (29.8)
Outside Kanagawa Prefecture	178 (9.2)	940 (8.8)

### Themes of unmet needs

In the qualitative analysis, as a result of slight differences between the needs-based themes we identified and the theme definitions proposed by Burg et al. [12], we made minor modifications to three of Burg et al.’s theme definitions (“communication,” “system of care,” and “cure”), and applied the remaining 13 directly. The modified points are shown in Additional file 1. Table 2 shows the modified three and the original 13 theme definitions and the frequency of occurrence of each need theme. The mean number of needs mentioned per consultation was 1.58 (standard deviation (SD) = 0.86; Table 3). Regarding types of cancers (Table 1), callers with multiple primary cancers had the largest mean number of needs 1.75 (SD = 1.20). Callers whose consultations were over 15 min in length had the highest mean number of needs 1.89 (SD = 1.04).

**Table 2:** Unmet needs themes (frequency and description)

	*n*	%	Codebook Description
1. Physical	391	20.2	Needs and issues experienced in or affecting the body, including pain, symptoms, sexual dysfunction, and care of body (such as diet, exercise, and rest).
2. Financial	86	4.4	Needs related to money, insurance, and the affordability of needed services and products.
3. Education/information	570	29.4	Needs related to unanswered questions and the lack of knowledge regarding what to expect as a cancer survivor, follow-up care, self-care, cancer and health research, and cancer risks, causes, and prevention.
4. Personal control	30	1.5	Needs related to an individual’s ability to maintain autonomy in terms of the physical self (sexual function, evacuation, and ambulation) and the social self (disclosure about cancer and ability to make plans and socialize). Also includes wishes to return to “normal” and finding a “new normal.”
5. System of care	76	3.9	Needs related to the health care system, including constraints, flaws, and limitations that affect early detection, diagnosis, treatment, follow-up care, continuity of care, and inadequate response from health care providers.
6. Resources	649	33.5	Needs related to availability and access to supplies, equipment, therapies and medications (including alternative and complementary), and transportation services.
7. Emotions/mental health	379	19.6	Needs related to psychological issues, including fear (recurrence, new cancers, death, and dying), depression, anxiety, and negative feelings (mistrust toward body, anger, and guilt).
8. Social support	30	1.5	Needs related to psychosocial and interpersonal issues, including intimacy, access to support groups, opportunities to use one’s own experiences to help others, and participation in social situations.
9. Societal	8	0.4	Needs revealed from respondents’ commentary about conditions and issues related to society’s response to cancer, including social norms, discrimination, misinformation, policies, and resource allocation (insurance coverage).
10. Communication	148	7.6	Needs related to engaging in discourse (talking) and information exchange (explaining) with others (including patients and doctors and patients and caregivers) and medical providers regarding cancer, cancer experience, and reconciliation with others.
11. Provider relationship	179	9.2	Needs related to trust in health care providers, including decision-making, follow-through, follow-up, and support.
12. Cure	474	24.5	Needs related to a wish for a cure for cancer and hopes for effective treatments (including through alternative medicine) for one’s self and for others.
13. Body image	7	0.4	Needs related to negative perception of body, including feeling unattractive and/or ashamed and loss of trust in body.
14. Survivor identity	1	0.1	Pertains to the respondent either explicitly identifying or not identifying as a cancer survivor because the respondent does not like the term “survivor” or feels that he or she has not reached a specific milestone to be called a survivor (e.g., not still in treatment or living a specific number of years since the diagnosis).
15. Employment	28	1.4	Needs pertaining to maintaining or obtaining a source of income that is appropriate given the cancer experience.
16. Existential	11	0.6	Needs pertaining to attaining peace in life and spirituality and making sense or meaning of the cancer experience.

**Table 3:** Average number of unmet needs stratified by each characteristics

		n	Mean value	Standard Deviation	Median	Inter-quartile range
Total		1938	1.58	0.86	1.00	1.00–2.00
Caller’s sex	*p* = 0.647					
Male		637	1.56	0.83	1.00	1.00–2.00
Female		1300	1.59	0.87	1.00	1.00–2.00
Survivor’s sex	*p* = 0.140					
Male		719	1.65	0.89	1.00	1.00–2.00
Female		995	1.60	0.88	1.00	1.00–2.00
Caller’s age group	*p* = 0.334					
20–30		282	1.64	0.92	1.00	1.00–2.00
40–50		756	1.61	0.88	1.00	1.00–2.00
≥ 60		830	1.56	0.83	1.00	1.00–2.00
Survivor’s age group	*p* = 0.160					
< 40		140	1.49	0.78	1.00	1.00–2.00
40–59		479	1.58	0.85	1.00	1.00–2.00
60–69		459	1.66	0.94	1.00	1.00–2.00
≥ 70		473	1.63	0.86	1.00	1.00–2.00
Relationship with survivor	*p* = 0.0679					
Survivor		1054	1.53	0.81	1.00	1.00–2.00
Spouse		278	1.64	0.94	1.00	1.00–2.00
Child		361	1.71	0.96	1.00	1.00–2.00
Parent		54	1.57	0.81	1.00	1.00–2.00
Sibling		64	1.69	0.79	2.00	1.00–2.00
Other		124	1.47	0.80	1.00	1.00–2.00
Treatment course	*p* < 0.0001					
Never got		492	1.52	0.75	1.00	1.00–2.00
Ongoing		694	1.78	0.98	1.00	1.00–2.00
Completed		468	1.56	0.82	1.00	1.00–2.00
Symptom	*p* < 0.0001					
Yes		1149	1.66	0.92	1.00	1.00–2.00
No		726	1.48	0.77	1.00	1.00–2.00
Residence	*p* = 0.907					
A city designated by ordinance		1162	1.57	0.84	1.00	1.00–2.00
Within Kanagawa Prefecture		540	1.61	0.91	1.00	1.00–2.00
Outside Kanagawa Prefecture		178	1.60	0.86	1.00	1.00–2.00
Past consultation history at KCC^a^	*p* < 0.0001					
Yes		120	1.33	0.64	1.00	1.00–2.00
No		1818	1.60	0.87	1.00	1.00–2.00
Cancer type	*p* = 0.0004					
Breast		275	1.71	0.97	1.00	1.00–2.00
Lung		189	1.66	0.89	1.00	1.00–2.00
Colorectal		181	1.57	0.82	1.00	1.00–2.00
Stomach		156	1.63	0.89	1.00	1.00–2.00
Other		669	1.63	0.87	1.00	1.00–2.00
Multi primary cancer		59	1.75	1.20	1.00	1.00–2.00
Never diagnosed with cancer		409	1.34	0.63	1.00	1.00–2.00
Call duration	p < 0.0001					
< 15 min		1141	1.37	0.63	1.00	1.00–2.00
≥ 15 min		797	1.89	1.04	2.00	1.00–2.00

^a^Kanagawa Cancer Center

### Differences in specific cancer types in survivors

In the multivariate analysis, we excluded survivor’s sex as a result of its significant correlation (in the chi-square test, *p* < 0.001) with caller’s sex.

In the primary analysis, for callers who were survivors, there were no significant differences in needs frequency among cancer types, except that individuals who were never diagnosed with cancer had a lower frequency of “physical” and “financial” needs than did survivors with breast cancer (Table 4).

**Table 4:** Logistic regression analysis (survivors)

	Odds ratio (95% Confidence interval)					
	**Physical**	**Financial**	**Education/information**	**Personal Control**	**System of Care**	**Resources**
Sex						
Male (reference)						
Female	2.50 (1.54–4.07)*	0.52 (0.22–1.21)	0.79 (0.54–1.15)	1.95 (0.59–6.42)	1.19 (0.47–3.05)	0.62 (0.43–0.91)*
Age group (in years)						
< 40, 40–59, 60–69, ≥ 70	1.25 (1.00–1.57)	1.03 (0.70–1.51)	0.94 (0.78–1.13)	0.80 (0.46–1.39)	0.77 (0.49–1.22)	0.98 (0.81–1.18)
Cancer type						
Breast (reference)						
Colon	1.05 (0.52–2.10)	0.52 (0.15–1.81)	1.11 (0.59–2.08)	0.59 (0.07–5.12)	1.99 (0.44–9.08)	1.34 (0.70–2.60)
Lung	0.72 (0.31–1.69)	0.15 (0.02–1.31)	1.72 (0.87–3.43)	0.78 (0.09–7.02)	0.85 (0.09–7.93)	1.39 (0.67–2.87)
Stomach	0.96 (0.44–2.10)	0.27 (0.05–1.40)	1.27 (0.63–2.58)	1.94 (0.34–11.00)	1.56 (0.26–9.24)	1.49 (0.73–3.03)
Other	0.78 (0.45–1.33)	0.53 (0.20–1.35)	1.11 (0.69–1.81)	1.17 (0.35–3.95)	1.56 (0.47–5.20)	1.23 (0.74–2.04)
Multi primary	2.33 (0.85–6.43)	0.35 (0.04–2.94)	0.49 (0.16–1.53)	3.97 (0.67–23.40)	1.95 (0.21–18.40)	1.25 (0.47–3.31)
Never diagnosed with cancer	0.41 (0.17–0.97)*	0.25 (0.06–0.98)*	1.77 (1.00–3.13)	0.00 (0.00-Inf)	1.83 (0.47–7.20)	1.40 (0.77–2.54)
Treatment course						
Pretreatment (reference)						
Ongoing	5.08 (2.64–9.78)*	0.71 (0.32–1.59)	1.28 (0.85–1.93)	1.31 (0.43–3.96)	0.70 (0.25–1.98)	0.51 (0.34–0.78)*
Completed	5.35 (2.74–10.40)*	0.91 (0.41–2.06)	0.78 (0.52–1.18)	0.28 (0.05–1.47)	0.69 (0.26–1.80)	0.68 (0.46–1.01)
Residence						
CDO^a^ (reference)						
Within KP^b^	1.12 (0.74–1.70)	0.85 (0.42–1.73)	0.75 (0.53–1.07)	1.19 (0.44–3.22)	1.44 (0.64–3.25)	1.16 (0.82–1.65)
Outside KP^b^	0.22 (0.06–0.79)*	0.80 (0.18–3.58)	0.90 (0.45–1.78)	1.67 (0.34–8.25)	0.64 (0.08–5.12)	1.51 (0.78–2.95)
Symptom						
Yes (reference)						
No	8.69 (5.35–14.10)*	0.67 (0.35–1.29)	0.46 (0.33–0.64)*	0.99 (0.38–2.59)	0.79 (0.35–1.76)	0.69 (0.50–0.96)*
Past consultation history at KCC^c^						
Yes (reference)						
No	1.22 (0.65–2.28)	1.91 (0.75–4.81)	1.18 (0.68–2.06)	0.53 (0.07–4.16)	0.91 (0.21–4.00)	0.54 (0.27–1.07)
	**Emotions/Mental Health**	**Social Support**	**Communications**	**Provider Relationship**	**Cure**	**Employment**
Sex						
Male (reference)						
Female	1.60 (1.05–2.43)*	1.76 (0.38–8.23)	1.81 (0.80–4.08)	1.25 (0.72–2.17)	0.77 (0.51–1.15)	0.34 (0.10–1.21)
Age group (in years)						
< 40, 40–59, 60–69, ≥ 70	0.95 (0.78–1.16)	1.30 (0.69–2.47)	1.18 (0.82–1.70)	0.98 (0.75–1.29)	1.03 (0.84–1.25)	0.37 (0.20–0.67)*
Cancer type						
Breast (reference)						
Colon	0.83 (0.43–1.62)	0.00 (0.00-Inf)	1.28 (0.48–3.41)	1.96 (0.86–4.48)	1.03 (0.52–2.04)	0.00 (0.00-Inf)
Lung	0.73 (0.33–1.61)	0.00 (0.00-Inf)	0.46 (0.10–2.17)	1.88 (0.73–4.83)	1.46 (0.71–3.02)	0.27 (0.02–2.86)
Stomach	0.93 (0.45–1.93)	0.41 (0.04–4.15)	0.46 (0.10–2.20)	1.17 (0.41–3.31)	1.13 (0.53–2.42)	0.17 (0.02–1.86)
Other	0.91 (0.56–1.47)	0.43 (0.10–1.88)	0.63 (0.26–1.49)	1.16 (0.57–2.36)	1.14 (0.68–1.92)	0.61 (0.19–1.98)
Multi primary	0.89 (0.33–2.40)	2.03 (0.32–13.00)	2.00 (0.57–7.00)	1.84 (0.55–6.18)	0.98 (0.35–2.73)	0.00 (0.00-Inf)
Never diagnosed with cancer	1.52 (0.85–2.71)	0.27 (0.03–2.58)	0.31 (0.07–1.45)	1.43 (0.59–3.44)	1.01 (0.53–1.93)	0.00 (0.00-Inf)
Treatment course						
Pretreatment (reference)						
Ongoing	1.14 (0.72–1.79)	1.36 (0.32–5.78)	1.48 (0.66–3.31)	1.63 (0.86–3.09)	0.97 (0.63–1.48)	1.01 (0.29–3.55)
Completed	1.23 (0.79–1.91)	1.42 (0.31–6.46)	0.68 (0.26–1.73)	1.26 (0.66–2.42)	0.53 (0.34–0.82)*	2.72 (0.86–8.57)
Residence						
CDO^a^ (reference)						
Within KP^b^	1.18 (0.82–1.70)	0.30 (0.07–1.38)	1.20 (0.63–2.30)	1.18 (0.71–1.94)	1.20 (0.83–1.74)	1.03 (0.41–2.58)
Outside KP^b^	0.79 (0.35–1.79)	1.01 (0.12–8.62)	0.39 (0.05–3.02)	0.85 (0.29–2.54)	1.92 (0.98–3.75)	0.00 (0.00-Inf)
Symptom						
Yes (reference)						
No	1.78 (1.25–2.53)*	1.21 (0.41–3.61)	1.31 (0.70–2.46)	1.49 (0.92–2.42)	0.56 (0.40–0.79)*	1.59 (0.62–4.10)
Past consultation history at KCC^c^						
Yes (reference)						
No	0.85 (0.47–1.55)	0.00 (0.00-Inf)	1.13 (0.42–3.03)	0.62 (0.24–1.60)	0.31 (0.13–0.74)*	0.44 (0.05–3.48)

*p < 0.05, ^a^A city designated by official ordinance, ^b^Kanagawa prefecture, ^c^Kanagawa Cancer Center

### Effects of other factors on survivors

In the secondary analysis, it is noteworthy that survivors with symptoms showed a higher frequency of “physical” and “emotional/mental health” needs than did survivors without symptoms (Table 4). Moreover, survivors with symptoms showed a lower frequency of “education/information”, “resource”, and “cure” needs than did survivors without symptoms.

Other statistically significant results are as follows: about sex, female participants showed a higher frequency of “physical” and “emotional/mental health” needs and a lower frequency of “resources” needs than male participants. About age, older age groups showed a lower frequency of “employment” needs than did the younger age group. About treatment course, compared to survivors with pretreatment, survivors with treatment ongoing and completed showed a higher frequency of “physical” needs. Survivors with ongoing treatment had a lower frequency of “resources” needs. Those with completed treatment had a lower frequency of “cure” needs. Other results are in Table 4.

We also conducted the similar analysis in callers except survivors (Additional file 1 and Table A1), and performed three additional, though slightly modified, logistic regression analyses. The results of the additional analyses are shown in Additional file 1, Table A2, A3, A4, A5 and A6.

## Discussion

### Small effects of specific cancer types

The primary findings of this study are that cancer type (and cancer site) has a small effect on survivors’ unmet needs and that their backgrounds—such as survivors’ age, sex, treatment course, and presence of symptoms—play more important roles in this regard as independent factors. This result is not consistent with those of previous studies, which suggested strong relationships between specific cancer types and specific unmet needs. For example, several studies have reported that breast-cancer survivors tend to have psychosocial needs [12, 18–20].

While cancer type may be merely a confounding factor for the influence of other backgrounds, three explanations for this inconsistency must be considered.

First, in this multivariate analysis, only the frequency of occurrence of each need was analyzed; the strength of each need was not. Therefore, the effect of strength might be underestimated in our results. Consequently, further studies that measure strength of needs are required. “The Supportive Care Needs Survey” is worth investigating in this regard [35].

Second, longer disease duration, which is usually related to type of cancer, might foster more serious “financial,” “personal control,” “social support,” and “employment” problems, as reported in previous studies [36–38]. The low frequency of these needs in this study might have resulted in insufficient statistical power to detect the impact of differing cancer types. There is a possibility that cancer types and sites affect needs, as a result of their influence on disease durations, but some needs themes may have been insufficiently frequent to show statistically significant differences in this regard.

Third, in clinical practice, different cancers cause different symptoms; for example, lymph edema after axillary lymph node dissection for breast cancer, decreased respiratory function after lung-cancer dissection, gastrointestinal obstruction for digestive cancer, and hot flashes and sexual function disorders as a result of hormone therapy. In this study, all needs regarding symptoms were integrated into the “physical” needs theme. This might have resulted in an underestimation of the differences among cancer types.

Further, this study indicated that differing needs among cancer types might be an indication of differences in people’s backgrounds in regard to major needs such as “education/information,” “resources,” and “emotional/mental health,” which we considered to have a sufficient frequency for analysis. As a result, we were able to examine these needs based on survivors’ individual backgrounds. However, for less-frequent needs themes and the “physical” theme (as mentioned above), we were unable to draw definite conclusions. Consequently, future large-scale studies are needed.

### Other interpretations of the results

There are more interpretations that are worth considering. Older survivors seem to have more physical problems, since they may have age-related decline in physical function and frailness. Moreover, they also seem to have lesser needs in terms of employment given their retirement from work. The findings support the hypothesis that older survivors tend to have more “physical” needs, while they have less “employment” needs.

Given the lack of enough data in this study, future studies should explore other possibilities based on the findings. For example, although previous studies have shown the relationships among sexual needs and cancer survivor’s background (sex and age) [39, 40], the present study could not consider this sexual needs, as those are rarely discussing in telephone consultations. This is likely because it is not common to talk about sexual concerns publicly in Japan. This in itself may indicate a social barrier and further research is needed to gain a better understanding. Further, although the relationships between emotional/mental health needs and social network have also been reported in a previous study [41], survivor/caregivers’ social support was not measured in the present study because of the passivity from the telephone consultation service. In our study, “emotional mental health” need was observed in 7 out of 16 cases (43%) of survivor/caregivers who had “social support” needs. We performed a Fisher’s exact test for the presence of these needs and found no significant difference (*p* = 0.056). Future studies should investigate this in more detail.

### Limitations

This study had some limitations. First, available data were obtained from telephone consultations; in these consultations, all calls were instigated in a spontaneous manner by survivors or caregivers. As a result, we could not evaluate unmet needs that were not mentioned in the consultations, or the needs of people who did not receive a consultation. This may have led to a selection bias, for some deference of results such as relationships between unmet needs and age, in contrast to a previous study, which included general patient population, although the proportions of sex and age of the sample were similar to those in our study [12]. In a study by Burg et al., the most frequent theme of unmet needs was “physical,” followed by “financial” and “education/information,” while in the present study, the most frequent one was “resources,” followed by “education/information” and “cure.” This suggests that the findings of the present study may have been strongly affected by the nature of telephone consultations as avenues to acquire information. In addition, approaching telephone consultation requires activeness, and individual personality traits may have affected the distribution of unmet needs in the present study. A previous study showed that personality traits may influence one’s ability to cope with living with cancer [42].

Second, we excluded repeat callers’ consultations from our analysis. Therefore, we may not have obtained an accurate evaluation of individuals who have many and severe unmet needs. Nevertheless, as the purpose of this study was to clarify what unmet needs survivors and their caregivers generally had; individuals who have many and severe unmet needs should be studied in the future.

Third, the qualitative data we analyzed were summaries recorded by each consultant, and these were not recorded word-for-word. Consequently, detailed context might have been lost, or may have been affected by the perspectives of the consultants.

Finally, some minor themes appeared too infrequently to be examined sufficiently; large-scale research that can investigate such minor themes is consequently needed.

## Conclusions

In conclusion, this study showed that cancer type was not a significant factor for specific unmet needs, and that individuals’ backgrounds and presence of symptoms play a more important role. Through this study, it was found that instruments to predict people’s needs and a system to provide individualized cancer care across cancer types should be developed in the future.

## Supplementary information

**Additional file 1.** Additional file. Supplementary result.

**Additional file 2:** Table A1. Logistic regression analysis (except survivors, adjusted by cancer type).

**Additional file 3:** Table A2. Logistic regression analysis (survivors, adjusted by specific cancer cite).

**Additional file 4:** Table A3. Logistic regression analysis (except survivors, adjusted by specific cancer cite).

**Additional file 5:** Table A4. Stratified logistic regression analysis 2006–2010 (survivors).

**Additional file 6:** Table A5. Stratified logistic regression analysis 2011–2014 (survivors).

**Additional file 7:** Table A6. Logistic regression analysis (including both survivors and caregivers).

## Data Availability

The datasets used and/or analyzed during the current study are available from the corresponding author on reasonable request.

## Abbreviations

KCCRI: Kanagawa Cancer Center Research Institute
KCCRIO: Kanagawa Cancer Clinical Research Information Organization
SD: Standard deviation
OR: Odds ratio

## References

[1] Cleeland CS (2007). Symptom burden: multiple symptoms and their impact as patient-reported outcomes. J Natl Cancer Inst Monogr.

[2] Amir Z, Wilson K, Hennings J, Young A (2012). The meaning of cancer: implications for family finances and consequent impact on lifestyle, activities, roles and relationships. Psychooncology.

[3] Boyes AW, Girgis A, D'Este C, Zucca AC (2012). Prevalence and correlates of cancer survivors' supportive care needs 6 months after diagnosis: a population-based cross-sectional study. BMC Cancer.

[4] Nakanotani T, Akechi T, Takayama T, Karato A, Kikuuchi Y, Okamoto N, Katayama K, Yokoo M, Ogawa A (2014). Characteristics of elderly cancer patients' concerns and their quality of life in Japan: a web-based survey. Jpn J Clin Oncol.

[5] Sklenarova H, Krumpelmann A, Haun MW, Friederich HC, Huber J, Thomas M, Winkler EC, Herzog W, Hartmann M (2015). When do we need to care about the caregiver? Supportive care needs, anxiety, and depression among informal caregivers of patients with cancer and cancer survivors. Cancer.

[6] Fennell KM, Heckel L, Wilson C, Byrnes M, Livingston PM (2016). How calls from carers, friends and family members of someone affected by cancer differ from those made by people diagnosed with cancer; analysis of 4 years of south Australian Cancer council helpline data. Support Care Cancer.

[7] Kaminska M, Kubiatowski T, Ciszewski T, Czarnocki KJ, Makara-Studzinska M, Bojar I, Staroslawska E (2015). Evaluation of symptoms of anxiety and depression in women with breast cancer after breast amputation or conservation treated with adjuvant chemotherapy. Ann Agric Environ Med.

[8] Pitceathly C, Maguire P (2003). The psychological impact of cancer on patients' partners and other key relatives: a review. Eur J Cancer.

[9] Friethriksdottir N, Saevarsdottir T, Halfdanardottir SI, Jonsdottir A, Magnusdottir H, Olafsdottir KL, Guethmundsdottir G, Gunnarsdottir S (2011). Family members of cancer patients: needs, quality of life and symptoms of anxiety and depression. Acta Oncol.

[10] Knapp S, Marziliano A, Moyer A (2014). Identity threat and stigma in cancer patients. Health Psychol Open.

[11] Lebel S, Devins GM (2008). Stigma in cancer patients whose behavior may have contributed to their disease. Future Oncol.

[12] Burg MA, Adorno G, Lopez ED, Loerzel V, Stein K, Wallace C, Sharma DK (2015). Current unmet needs of cancer survivors: analysis of open-ended responses to the American Cancer Society study of Cancer survivors II. Cancer.

[13] Miedema B, Easley J, Robinson LM (2013). Do current cancer follow-up care practices meet the needs of young adult cancer survivors in Canada? A qualitative inquiry. Curr Oncol.

[14] Ross L, Petersen MA, Johnsen AT, Lundstrom LH, Groenvold M (2013). Cancer patients' evaluation of communication: a report from the population-based study 'The Cancer Patient's World'. Support Care Cancer.

[15] Wiggers JH, Donovan KO, Redman S, Sanson-Fisher RW (1990). Cancer patient satisfaction with care. Cancer.

[16] Sanson-Fisher R, Girgis A, Boyes A, Bonevski B, Burton L, Cook P (2000). The unmet supportive care needs of patients with cancer. Supportive Care Review Group. Cancer.

[17] Armes J, Crowe M, Colbourne L, Morgan H, Murrells T, Oakley C, Palmer N, Ream E, Young A, Richardson A (2009). Patients' supportive care needs beyond the end of cancer treatment: a prospective, longitudinal survey. J Clin Oncol.

[18] Gooden HM, White KJ (2013). Pancreatic cancer and supportive care--pancreatic exocrine insufficiency negatively impacts on quality of life. Support Care Cancer.

[19] Salander P, Isaksson J, Granstrom B, Laurell G (2016). Motives that head and neck cancer patients have for contacting a specialist nurse - an empirical study. J Clin Nurs.

[20] Uchida M, Akechi T, Okuyama T, Sagawa R, Nakaguchi T, Endo C, Yamashita H, Toyama T, Furukawa TA (2011). Patients' supportive care needs and psychological distress in advanced breast cancer patients in Japan. Jpn J Clin Oncol.

[21] Marcus AC, Garrett KM, Cella D, Wenzel L, Brady MJ, Fairclough D, Pate-Willig M, Barnes D, Emsbo SP, Kluhsman BC (2010). Can telephone counseling post-treatment improve psychosocial outcomes among early stage breast cancer survivors?. Psychooncology.

[22] Gotay CC, Bottomley A (1998). Providing psycho-social support by telephone: what is its potential in cancer patients?. Eur J Cancer Care (Engl).

[23] Kinney AY, Steffen LE, Brumbach BH, Kohlmann W, Du R, Lee JH, Gammon A, Butler K, Buys SS, Stroup AM (2016). Randomized noninferiority trial of telephone delivery of BRCA1/2 genetic counseling compared with in-person counseling: 1-year follow-up. J Clin Oncol.

[24] Stacey D, Chambers SK, Jacobsen MJ, Dunn J (2008). Overcoming barriers to cancer-helpline professionals providing decision support for callers: an implementation study. Oncol Nurs Forum.

[25] Reid J, Porter S (2011). Utility, caller, and patient profile of a novel chemotherapy telephone helpline service within a regional cancer Centre in Northern Ireland. Cancer Nurs.

[26] Lin W-L, Sun J-L, Chang S-C, Wu P-H, Tsai T-C, Huang W-T, Tsao C-J (2014). Development and application of telephone counseling Services for Care of patients with colorectal Cancer. Asian Pac J Cancer Prev.

[27] Chambers SK, Girgis A, Occhipinti S, Hutchison S, Turner J, Morris B, Dunn J (2012). Psychological distress and unmet supportive care needs in cancer patients and carers who contact cancer helplines. Eur J Cancer Care (Engl).

[28] Morris BA, Thorndike FP, Ritterband LM, Glozier N, Dunn J, Chambers SK (2015). Sleep disturbance in cancer patients and caregivers who contact telephone-based help services. Support Care Cancer.

[29] Marcus AC, Garrett KM, Kulchak-Rahm A, Barnes D, Dortch W, Juno S (2002). Telephone counseling in psychosocial oncology: a report from the Cancer information and counseling line. Patient Educ Couns.

[30] Jefford M, Black C, Grogan S, Yeoman G, White V, Akkerman D (2005). Information and support needs of callers to the Cancer helpline, the Cancer Council Victoria. Eur J Cancer Care (Engl).

[31] Hardyman R, Hardy P, Brodie J, Stephens R (2005). It's good to talk: comparison of a telephone helpline and website for cancer information. Patient Educ Couns.

[32] Powe BD, Nehl EJ, Blanchard CM, Finnie R (2005). Meeting the public's cancer information needs: characteristics of callers to the National Cancer Information Center of the American Cancer Society. J Cancer Educ.

[33] Shapiro CL (2018). Cancer survivorship. N Engl J Med.

[34] Kanda Y (2013). Investigation of the freely available easy-to-use software 'EZR' for medical statistics. Bone Marrow Transplant.

[35] Bonevski B, Sanson-Fisher R, Girgis A, Burton L, Cook P, Boyes A (2000). Evaluation of an instrument to assess the needs of patients with cancer. Supportive Care Review Group. Cancer.

[36] Nipp RD, Kirchhoff AC, Fair D, Rabin J, Hyland KA, Kuhlthau K, Perez GK, Robison LL, Armstrong GT, Nathan PC (2017). Financial burden in survivors of childhood Cancer: a report from the childhood Cancer survivor study. J Clin Oncol.

[37] Dura-Ferrandis E, Mandelblatt JS, Clapp J, Luta G, Faul L, Kimmick G, Cohen HJ, Yung RL, Hurria A (2017). Personality, coping, and social support as predictors of long-term quality-of-life trajectories in older breast cancer survivors: CALGB protocol 369901 (Alliance). Psychooncology.

[38] Stone DS, Ganz PA, Pavlish C, Robbins WA (2017). Young adult cancer survivors and work: a systematic review. J Cancer Surviv.

[39] Ledebo A, Bock D, Prytz M, Haglind E, Angenete E (2018). Urogenital function 3 years after abdominoperineal excision for rectal cancer. Color Dis.

[40] Akechi T, Okuyama T, Uchida M, Nakaguchi T, Ito Y, Yamashita H, Toyama T, Komatsu H, Kizawa Y, Wada M (2012). Perceived needs, psychological distress and quality of life of elderly cancer patients. Jpn J Clin Oncol.

[41] Stinesen Kollberg K, Thorsteinsdottir T, Wilderang U, Hugosson J, Wiklund P, Bjartell A, Carlsson S, Stranne J, Haglind E, Steineck G (2018). Social constraints and psychological well-being after prostate cancer: a follow-up at 12 and 24 months after surgery. Psychooncology.

[42] Kenne Sarenmalm E, Browall M, Persson LO, Fall-Dickson J, Gaston-Johansson F (2013). Relationship of sense of coherence to stressful events, coping strategies, health status, and quality of life in women with breast cancer. Psychooncology.

