# Supplementary material for: Impact of individual background on the unmet needs of cancer survivors and caregivers – a mixed-methods analysis
Source: BMC Cancer. 2020 Mar 30;20:263. doi: 10.1186/s12885-020-06732-5 (PMC7106842; doi:10.1186/s12885-020-06732-5)
Supplement: Supplementary file 1 — Additional file 1. Additional file. Supplementary result. [file 12885_2020_6732_MOESM1_ESM.docx]

Additional file: supplementary result

Modification of the theme

The original “communication” theme was defined as “needs related to discourse (talking) and information exchange (explaining) about cancer and cancer experience with others (including survivor and doctor, and survivor and family/friends/employers) and among medical providers.” However, we included in the theme, the needs regarding conflicts and constraints within the family. For instance, in the data we analyzed a daughter of a survivor with lung cancer who said, “my father says that as long as he feels less pain, he is satisfied, even if he is not receiving life-prolonging treatment. But his sister wishes him to live as long as possible.” The original “system of care” theme was defined as, “needs related to the health care system, including constraints and flaws that affect early detection, diagnosis, treatment, follow-up care, continuity of care, and inadequate response from health care providers.” Here, we incorporated into the theme needs regarding problems that the current Japanese medical system cannot solve. For example, the wife of a patient who had died from gastric cancer said, “my husband died from brain metastasis of gastric cancer, but even now I can’t help wondering if he could have been treated earlier.” The original “cure” theme was defined as “needs related to a wish for a cure for cancer and hopes of effective treatments for self and others.” Here, we included in this theme needs relating to placing hope in alternative medicine. For example, a son of a survivor who had lung cancer said, “my mother has received chemotherapy for several months, but it has not shown any obvious effect. I am considering sourcing immunotherapy or cancer vaccination for her.”

Supplementary result of multivariate analysis

1. Analysis in callers except survivors.

In the primary analysis, among callers who were not cancer survivors (i.e. the caregivers), callers for colorectal cancer survivors had a lower frequency of “emotional/mental health” needs than did callers for breast-cancer survivors, but this was the only significant difference in needs frequency among callers for survivors with specific cancer types (Table A1). The results of secondary analysis (other factors) was also shown in Table A1.

1. Analysis with specific cancer site

We conducted additionally analysis that “cancer type” replaced with “specific cancer site” as an independent variable (digestive, breast, respiratory, urological, and gynecological). Consequently, we determined that when the caller was a survivor, survivors with breast cancer and gynecological cancer showed a higher frequency of “employment” needs than survivors with digestive cancer (Table A2). When the caller was not a survivor, callers for gynecological cancer survivors showed a higher frequency of “provider relationship” needs than callers for digestive cancer survivors (Table A3). Callers who were never diagnosed with cancer showed a higher frequency of “emotional/mental health” needs than callers for digestive-cancer survivors.

1. Difference between survivors and caregivers

As an additional analysis, we performed a multivariable logistic regression analysis to compare survivors and caregivers, since some studies have reported differences in unmet needs between survivors and caregivers, although in our knowledge, no study has considered the effect of each group’s background.

We found that caregivers showed a higher frequency of “resources,” “communication,” and “cure” needs, and a lower frequency of “physical,” “personal control,” and “employment” needs than did survivors (Table A6).

1. No difference in attitude between eras

This study was based on data collected from October 2006 to May 2014. During this long period, changes in survivors and care-providers attitudes toward needs and cancer care may have occurred. Therefore, cancer care and time might have been potential confounders. However, the results of the stratified analysis, which divided time into 2006-2010 and 2011-2014, showed no critical discrepancy with the main multi variated analysis (Tables A4 and A5).
