# Supplementary material for: Impact of individual background on the unmet needs of cancer survivors and caregivers – a mixed-methods analysis
Source: BMC Cancer. 2020 Mar 30;20:263. doi: 10.1186/s12885-020-06732-5 (PMC7106842; doi:10.1186/s12885-020-06732-5)
Supplement: Supplementary file 2 — Additional file 2: Table A1. Logistic regression analysis (except survivors, adjusted by cancer type). [file 12885_2020_6732_MOESM2_ESM.docx]

| **Table A1. Logistic regression analysis (all callers except survivor)** | | | | | | | |
| --- | --- | --- | --- | --- | --- | --- | --- |
|  | Odds ratio (95% Confidence interval) | | | | | |  |
|  | Physical | Financial | Education/Information | Personal Control | System of Care | Resources |  |
| Caller's sex |  |  |  |  |  |  |  |
| Male(reference) |  |  |  |  |  |  |  |
| Female | 1.60 (0.85-3.01) | 1.61 (0.52-5.01) | 0.72 (0.45-1.15) | 0.97 (0.15-6.14) | 1.38 (0.48-3.95) | 0.93 (0.59-1.45) |  |
| Caller's age group (in years) |  |  |  |  |  |  |  |
| < 40, 40-59, ≥ 60 | 0.64 (0.36-1.13) | 1.63 (0.62-4.31) | 1.12 (0.74-1.70) | 0.50 (0.10-2.44) | 1.50 (0.59-3.81) | 1.13 (0.76-1.68) |  |
| Survivor's age group (in years) |  |  |  |  |  |  |  |
| < 40, 40-59, 60-69, ≥ 70 | 1.71 (1.11-2.64)* | 0.64 (0.30-1.37) | 0.85 (0.61-1.17) | 0.86 (0.28-2.71) | 1.52 (0.71-3.27) | 1.10 (0.81-1.50) |  |
| Cancer type |  |  |  |  |  |  |  |
| Breast (reference) |  |  |  |  |  |  |  |
| Colon | 0.51 (0.17-1.51) | 5.47 (0.50-59.50) | 0.59 (0.24-1.46) | 0.39 (0.02-8.52) | 0.86 (0.17-4.26) | 1.32 (0.55-3.18) |  |
| Lung | 0.48 (0.17-1.38) | 0.00 (0.00-Inf) | 1.06 (0.45-2.53) | 0.84 (0.06-12.50) | 1.16 (0.26-5.06) | 1.40 (0.59-3.33) |  |
| Stomach | 0.50 (0.15-1.62) | 4.86 (0.41-57.90) | 0.80 (0.32-2.01) | 0.00 (0.00-Inf) | 0.00 (0.00-Inf) | 1.25 (0.50-3.13) |  |
| Other | 0.43 (0.17-1.09) | 3.66 (0.40-33.60) | 0.69 (0.33-1.44) | 0.32 (0.02-4.36) | 0.36 (0.08-1.58) | 1.44 (0.68-3.06) |  |
| Multi primary | 0.72 (0.17-3.09) | 0.00 (0.00-Inf) | 0.58 (0.14-2.49) | 0.00 (0.00-Inf) | 1.00 (0.13-7.56) | 1.01 (0.27-3.71) |  |
| Never diagnosed with cancer | 1.87 (0.39-8.94) | 0.00 (0.00-Inf) | 0.86 (0.28-2.59) | 0.00 (0.00-Inf) | 0.00 (0.00-Inf) | 0.71 (0.23-2.25) |  |
| Relationship with survivor |  |  |  |  |  |  |  |
| Spouse (reference) |  |  |  |  |  |  |  |
| Child | 0.33 (0.14-0.78)* | 1.20 (0.24-6.05) | 1.41 (0.69-2.87) | 0.00 (0.00-Inf) | 2.14 (0.50-9.13) | 1.28 (0.66-2.49) |  |
| Parent | 0.18 (0.02-1.76) | 2.47 (0.42-14.60) | 0.71 (0.24-2.09) | 0.00 (0.00-Inf) | 3.19 (0.21-48.70) | 0.56 (0.18-1.71) |  |
| Sibling | 0.32 (0.10-1.06) | 0.42 (0.05-3.95) | 1.14 (0.46-2.82) | 0.72 (0.07-7.29) | 0.00 (0.00-Inf) | 0.89 (0.39-2.07) |  |
| Other | 0.33 (0.11-1.02) | 0.46 (0.05-4.20) | 1.24 (0.55-2.77) | 0.55 (0.04-7.69) | 1.49 (0.25-9.05) | 0.77 (0.35-1.70) |  |
| Treatment course |  |  |  |  |  |  |  |
| Pretreatment (reference) |  |  |  |  |  |  |  |
| Ongoing | 4.70 (2.10-10.50)* | 1.64 (0.53-5.04) | 1.28 (0.77-2.12) | 2.01 (0.21-19.60) | 1.12 (0.36-3.53) | 0.73 (0.46-1.17) |  |
| Completed | 4.83 (2.01-11.60)* | 0.71 (0.12-4.06) | 0.71 (0.37-1.34) | 0.94 (0.05-18.20) | 2.10 (0.65-6.85) | 0.68 (0.39-1.19) |  |
| Residence |  |  |  |  |  |  |  |
| CDO† (reference) |  |  |  |  |  |  |  |
| Within KP‡ | 1.08 (0.61-1.89) | 1.34 (0.49-3.67) | 1.03 (0.64-1.67) | 2.00 (0.35-11.30) | 1.41 (0.56-3.52) | 1.03 (0.66-1.61) |  |
| Outside KP‡ | 0.55 (0.22-1.37) | 0.52 (0.06-4.36) | 1.29 (0.70-2.41) | 0.00 (0.00-Inf) | 1.34 (0.37-4.79) | 1.45 (0.81-2.61) |  |
| Symptom |  |  |  |  |  |  |  |
| Yes (reference) |  |  |  |  |  |  |  |
| No | 9.82 (3.69-26.10)* | 0.73 (0.26-2.04) | 0.69 (0.43-1.11) | 0.92 (0.16-5.34) | 1.81 (0.58-5.65) | 0.83 (0.52-1.30) |  |
| Past consultation history at KCC§ |  |  |  |  |  |  |  |
| Yes (reference) |  |  |  |  |  |  |  |
| No | 3.81 (1.26-11.50)* | 2.14 (0.43-10.70) | 1.25 (0.46-3.41) | 0.00 (0.00-Inf) | 0.00 (0.00-Inf) | 0.17 (0.04-0.76)* |  |
| *p < 0.05, †A city designated by official ordinance, ‡ Kanagawa prefecture, §Kanagawa Cancer Center | | | | | | | |

| **Table A1. Logistic regression analysis (all callers except survivor, continued)** | | | | | | |
| --- | --- | --- | --- | --- | --- | --- |
|  | Odds ratio (95% Confidence interval) | | | | | |
|  | Emotions/Mental Health | Social Support | Communications | Provider Relationship | Cure | Employment |
| Caller's sex |  |  |  |  |  |  |
| Male(reference) |  |  |  |  |  |  |
| Female | 1.59 (0.90-2.84) | 2.61 (0.29-23.30) | 1.41 (0.71-2.80) | 0.53 (0.27-1.02) | 0.76 (0.48-1.20) | 0.33 (0.03-3.21) |
| Caller's age group (in years) |  |  |  |  |  |  |
| < 40, 40-59, ≥ 60 | 0.85 (0.52-1.37) | 0.41 (0.08-2.06) | 0.82 (0.49-1.38) | 0.78 (0.40-1.50) | 0.62 (0.41-0.95)* | 0.12 (0.00-4.10) |
| Survivor's age group (in years) |  |  |  |  |  |  |
| < 40, 40-59, 60-69, ≥ 70 | 0.74 (0.51-1.08) | 1.48 (0.47-4.64) | 0.92 (0.61-1.38) | 1.13 (0.68-1.88) | 1.28 (0.92-1.77) | 3.05 (0.23-41.20) |
| Cancer type |  |  |  |  |  |  |
| Breast (reference) |  |  |  |  |  |  |
| Colon | 0.31 (0.11-0.88)* | 0.66 (0.00-Inf) | 0.62 (0.18-2.17) | 0.58 (0.13-2.57) | 1.02 (0.43-2.43) | 1.40 (0.00-Inf) |
| Lung | 0.48 (0.18-1.26) | 283 x10^5^ (0.00-Inf) | 0.50 (0.14-1.85) | 0.50 (0.10-2.44) | 0.66 (0.28-1.59) | 573 x10^6^ (0.00-Inf) |
| Stomach | 0.74 (0.28-2.00) | 302 x10^5^ (0.00-Inf) | 1.00 (0.28-3.55) | 2.12 (0.55-8.25) | 0.51 (0.20-1.29) | 539 x10^6^ (0.00-Inf) |
| Other | 0.55 (0.25-1.20) | 327 x10^5^ (0.00-Inf) | 1.06 (0.40-2.82) | 1.47 (0.47-4.61) | 0.94 (0.45-1.95) | 123 x10^6^ (0.00-Inf) |
| Multi primary | 0.17 (0.02-1.48) | 0.42 (0.00-Inf) | 1.98 (0.44-8.83) | 1.41 (0.22-9.19) | 0.73 (0.20-2.67) | 16.40 (0.00-Inf) |
| Never diagnosed with cancer | 2.55 (0.85-7.63) | 1.30 (0.00-Inf) | 0.71 (0.15-3.42) | 1.73 (0.32-9.47) | 0.43 (0.14-1.35) | 0.25 (0.00-Inf) |
| Relationship with survivor |  |  |  |  |  |  |
| Spouse (reference) |  |  |  |  |  |  |
| Child | 0.56 (0.26-1.24) | 0.17 (0.01-2.29) | 1.12 (0.44-2.89) | 1.03 (0.35-3.10) | 0.81 (0.41-1.64) | 0.00 (0.00-Inf) |
| Parent | 0.51 (0.16-1.59) | 0.00 (0.00-Inf) | 2.62 (0.74-9.33) | 0.99 (0.20-5.03) | 1.59 (0.56-4.54) | 10.10 (0.09-1090.00) |
| Sibling | 0.63 (0.24-1.63) | 0.00 (0.00-Inf) | 1.49 (0.47-4.75) | 1.66 (0.47-5.90) | 0.99 (0.42-2.34) | 4.86 (0.15-155.00) |
| Other | 0.35 (0.13-0.97)* | 0.80 (0.05-13.40) | 1.98 (0.71-5.55) | 0.55 (0.14-2.24) | 0.56 (0.24-1.30) | 0.00 (0.00-Inf) |
| Treatment course |  |  |  |  |  |  |
| Pretreatment (reference) |  |  |  |  |  |  |
| Ongoing | 1.60 (0.88-2.90) | 1.75 (0.17-17.90) | 1.58 (0.77-3.22) | 2.20 (0.99-4.86) | 0.94 (0.58-1.53) | 0.30 (0.03-2.86) |
| Completed | 1.49 (0.74-3.01) | 4.06 (0.40-41.00) | 1.75 (0.76-4.01) | 0.94 (0.33-2.65) | 0.82 (0.46-1.46) | 0.00 (0.00-Inf) |
| Residence |  |  |  |  |  |  |
| CDO† (reference) |  |  |  |  |  |  |
| Within KP‡ | 0.83 (0.48-1.43) | 0.58 (0.10-3.38) | 0.71 (0.37-1.36) | 0.51 (0.24-1.10) | 1.29 (0.82-2.03) | 1.72 (0.15-19.60) |
| Outside KP‡ | 1.12 (0.55-2.29) | 0.93 (0.09-9.21) | 0.45 (0.16-1.24) | 0.40 (0.13-1.23) | 1.81 (1.00-3.30) | 0.00 (0.00-Inf) |
| Symptom |  |  |  |  |  |  |
| Yes (reference) |  |  |  |  |  |  |
| No | 1.31 (0.74-2.32) | 2.57 (0.29-22.70) | 1.20 (0.61-2.37) | 0.87 (0.42-1.79) | 0.64 (0.41-1.01) | 0.79 (0.05-11.60) |
| Past consultation history at KCC§ |  |  |  |  |  |  |
| Yes (reference) |  |  |  |  |  |  |
| No | 0.58 (0.16-2.15) | 0.00 (0.00-Inf) | 0.60 (0.13-2.79) | 0.37 (0.05-2.93) | 0.09 (0.01-0.66)* | 0.00 (0.00-Inf) |
| *p < 0.05, †A city designated by official ordinance, ‡ Kanagawa prefecture, §Kanagawa Cancer Center | | | | | | |
