# Supplementary material for: Impact of individual background on the unmet needs of cancer survivors and caregivers – a mixed-methods analysis
Source: BMC Cancer. 2020 Mar 30;20:263. doi: 10.1186/s12885-020-06732-5 (PMC7106842; doi:10.1186/s12885-020-06732-5)
Supplement: Supplementary file 3 — Additional file 3: Table A2. Logistic regression analysis (survivors, adjusted by specific cancer cite). [file 12885_2020_6732_MOESM3_ESM.docx]

| **Table A2. Logistic regression analysis (survivors)** | | | | | | |
| --- | --- | --- | --- | --- | --- | --- |
|  | Odds ratio (95% Confidence interval) | | | | | |
|  | Physical | Financial | Education/Information | Personal Control | System of Care | Resources |
| Sex |  |  |  |  |  |  |
| Male (reference) |  |  |  |  |  |  |
| Female | 2.44 (1.44-4.15)* | 0.40 (0.15-1.07) | 0.84 (0.56-1.27) | 1.86 (0.47-7.41) | 1.14 (0.42-3.08) | 0.63 (0.42-0.95)* |
| Age group (in years) |  |  |  |  |  |  |
| < 40, 40-59, 60-69, ≥ 70 | 1.31 (1.04-1.64)* | 1.07 (0.73-1.56) | 0.91 (0.75-1.09) | 0.88 (0.51-1.51) | 0.76 (0.48-1.21) | 0.98 (0.81-1.18) |
| Specific cancer site |  |  |  |  |  |  |
| Digestive (reference) |  |  |  |  |  |  |
| Breast | 1.02 (0.57-1.82) | 3.05 (0.95-9.83) | 0.78 (0.46-1.31) | 0.95 (0.23-3.99) | 0.37 (0.11-1.30) | 0.74 (0.42-1.29) |
| Respiratory | 0.65 (0.29-1.50) | 0.39 (0.05-3.22) | 1.35 (0.71-2.56) | 0.69 (0.07-6.47) | 0.30 (0.04-2.44) | 1.10 (0.57-2.12) |
| Urologic | 0.64 (0.26-1.58) | 0.89 (0.28-2.87) | 0.90 (0.51-1.60) | 1.11 (0.18-7.00) | 0.19 (0.02-1.56) | 0.90 (0.51-1.59) |
| Gynecologic | 1.01 (0.47-2.19) | 2.42 (0.56-10.50) | 0.58 (0.28-1.23) | 1.36 (0.25-7.58) | 0.58 (0.13-2.54) | 0.93 (0.46-1.88) |
| Other | 0.87 (0.44-1.72) | 2.01 (0.67-6.03) | 1.00 (0.55-1.80) | 1.77 (0.37-8.43) | 0.38 (0.08-1.86) | 1.16 (0.65-2.07) |
| Never diagnosed with cancer | 0.42 (0.18-0.98)* | 0.68 (0.17-2.71) | 1.40 (0.82-2.42) | 0.00 (0.00-Inf) | 0.68 (0.20-2.26) | 1.07 (0.62-1.86) |
| Treatment course |  |  |  |  |  |  |
| Pretreatment (reference) |  |  |  |  |  |  |
| Ongoing | 5.27 (2.74-10.20)* | 0.75 (0.33-1.68) | 1.21 (0.81-1.83) | 1.37 (0.46-4.03) | 0.66 (0.24-1.84) | 0.51 (0.34-0.78)* |
| Completed | 5.41 (2.77-10.50)* | 0.90 (0.40-2.02) | 0.74 (0.49-1.12) | 0.28 (0.05-1.50) | 0.60 (0.23-1.59) | 0.67 (0.45-1.01) |
| Residence |  |  |  |  |  |  |
| CDO† (reference) |  |  |  |  |  |  |
| Within KP‡ | 1.14 (0.75-1.73) | 0.81 (0.40-1.65) | 0.74 (0.52-1.05) | 1.24 (0.46-3.34) | 1.52 (0.67-3.44) | 1.14 (0.81-1.62) |
| Outside KP‡ | 0.25 (0.07-0.88)* | 0.78 (0.18-3.44) | 0.91 (0.46-1.78) | 1.64 (0.33-8.21) | 0.67 (0.08-5.37) | 1.51 (0.77-2.93) |
| Symptom |  |  |  |  |  |  |
| Yes (reference) |  |  |  |  |  |  |
| No | 8.19 (5.07-13.20)* | 0.62 (0.32-1.19) | 0.47 (0.34-0.65)* | 0.96 (0.37-2.49) | 0.75 (0.34-1.69) | 0.69 (0.49-0.96)* |
| Past consultation history at KCC§ |  |  |  |  |  |  |
| Yes (reference) |  |  |  |  |  |  |
| No | 1.21 (0.65-2.26) | 1.81 (0.72-4.57) | 1.19 (0.68-2.08) | 0.54 (0.07-4.23) | 0.98 (0.22-4.34) | 0.54 (0.27-1.06) |
| **p* < 0.05, †A city designated by official ordinance, ‡Kanagawa prefecture, §Kanagawa Cancer Center | | | | | | |

| **Table A2. Logistic regression analysis (survivors, continued)** | | | | | | |
| --- | --- | --- | --- | --- | --- | --- |
|  | Odds ratio (95% Confidence interval) | | | | | |
|  | Emotions/  Mental Health | Social Support | Communications | Provider Relationship | Cure | Employment |
| Sex |  |  |  |  |  |  |
| Male (reference) |  |  |  |  |  |  |
| Female | 1.59 (1.01-2.52)* | 1.93 (0.37-10.10) | 2.15 (0.86-5.37) | 1.39 (0.76-2.55) | 0.69 (0.44-1.08) | 0.14 (0.02-0.99)* |
| Age group (in years) |  |  |  |  |  |  |
| < 40, 40-59, 60-69, ≥ 70 | 0.96 (0.79-1.17) | 1.41 (0.75-2.65) | 1.21 (0.84-1.73) | 0.98 (0.75-1.29) | 1.04 (0.85-1.27) | 0.40 (0.22-0.73)* |
| Specific cancer site |  |  |  |  |  |  |
| Digestive (reference) |  |  |  |  |  |  |
| Breast | 1.30 (0.76-2.24) | 3.16 (0.65-15.40) | 1.11 (0.46-2.69) | 0.64 (0.31-1.34) | 1.03 (0.58-1.83) | 10.40 (1.16-93.20)* |
| Respiratory | 0.89 (0.41-1.94) | 0.00 (0.00-Inf) | 0.51 (0.11-2.39) | 1.18 (0.49-2.85) | 1.60 (0.82-3.11) | 1.31 (0.12-14.00) |
| Urologic | 1.18 (0.58-2.42) | 1.15 (0.10-13.30) | 0.97 (0.28-3.42) | 0.97 (0.39-2.37) | 0.99 (0.55-1.80) | 2.02 (0.29-13.90) |
| Gynecologic | 1.06 (0.52-2.16) | 0.00 (0.00-Inf) | 0.77 (0.22-2.75) | 0.73 (0.28-1.93) | 1.47 (0.71-3.05) | 9.91 (0.95-103.00)* |
| Other | 1.58 (0.86-2.93) | 2.08 (0.33-13.10) | 0.81 (0.25-2.66) | 0.69 (0.28-1.69) | 0.95 (0.50-1.81) | 2.28 (0.42-12.40) |
| Never diagnosed with cancer | 1.95 (1.08-3.52)* | 0.77 (0.07-8.26) | 0.36 (0.07-1.74) | 0.93 (0.40-2.15) | 1.00 (0.54-1.84) | 0.00 (0.00-Inf) |
| Treatment course |  |  |  |  |  |  |
| Pretreatment (reference) |  |  |  |  |  |  |
| Ongoing | 1.14 (0.73-1.79) | 1.17 (0.28-4.89) | 1.64 (0.73-3.65) | 1.70 (0.90-3.22) | 0.98 (0.64-1.49) | 0.92 (0.26-3.24) |
| Completed | 1.24 (0.79-1.93) | 1.28 (0.28-5.85) | 0.73 (0.29-1.87) | 1.31 (0.68-2.52) | 0.54 (0.34-0.84)* | 2.50 (0.78-8.08) |
| Residence |  |  |  |  |  |  |
| CDO† (reference) |  |  |  |  |  |  |
| Within KP‡ | 1.16 (0.80-1.67) | 0.34 (0.07-1.54) | 1.22 (0.64-2.32) | 1.18 (0.72-1.94) | 1.21 (0.84-1.74) | 1.11 (0.45-2.78) |
| Outside KP‡ | 0.79 (0.35-1.78) | 1.33 (0.16-11.30) | 0.42 (0.05-3.22) | 0.88 (0.30-2.61) | 1.87 (0.96-3.66)* | 0.00 (0.00-Inf) |
| Symptom |  |  |  |  |  |  |
| Yes (reference) |  |  |  |  |  |  |
| No | 1.79 (1.26-2.54)* | 1.29 (0.44-3.79) | 1.25 (0.66-2.35) | 1.48 (0.91-2.41) | 0.55 (0.39-0.79)* | 1.59 (0.61-4.15) |
| Past consultation history at KCC§ |  |  |  |  |  |  |
| Yes (reference) |  |  |  |  |  |  |
| No | 0.84 (0.46-1.53) | 0.00 (0.00-Inf) | 1.11 (0.42-2.98) | 0.63 (0.24-1.62) | 0.31 (0.13-0.73)* | 0.39 (0.05-3.13) |
| **p* < 0.05, †A city designated by official ordinance, ‡Kanagawa prefecture, §Kanagawa Cancer Center | | | | | | |
