# Supplementary material for: Impact of individual background on the unmet needs of cancer survivors and caregivers – a mixed-methods analysis
Source: BMC Cancer. 2020 Mar 30;20:263. doi: 10.1186/s12885-020-06732-5 (PMC7106842; doi:10.1186/s12885-020-06732-5)
Supplement: Supplementary file 4 — Additional file 4: Table A3. Logistic regression analysis (except survivors, adjusted by specific cancer cite). [file 12885_2020_6732_MOESM4_ESM.docx]

| **Table A3. Logistic regression analysis (except survivor)** | | | | | | | |
| --- | --- | --- | --- | --- | --- | --- | --- |
|  | Odds ratio (95% Confidence interval) | | | | | |  |
|  | Physical | Financial | Education/Information" | Personal Control | System of Care | Resources |  |
| Caller's sex |  |  |  |  |  |  |  |
| Male(reference) |  |  |  |  |  |  |  |
| Female | 1.70 (0.90-3.22) | 1.48 (0.47-4.67) | 0.72 (0.45-1.16) | 1.56 (0.12-21.00) | 1.45 (0.51-4.12) | 0.87 (0.55-1.38) |  |
| Caller's age group (in years) |  |  |  |  |  |  |  |
| < 40, 40-59, ≥ 60 | 0.63 (0.36-1.11) | 2.06 (0.75-5.67) | 1.12 (0.73-1.71) | 0.44 (0.09-2.12) | 1.65 (0.67-4.07) | 1.16 (0.78-1.73) |  |
| Survivor's age group (in years) |  |  |  |  |  |  |  |
| < 40, 40-59, 60-69, ≥ 70 | 1.78 (1.15-2.76)* | 0.54 (0.25-1.19) | 0.86 (0.62-1.19) | 0.83 (0.27-2.54) | 1.47 (0.70-3.10) | 1.04 (0.76-1.43) |  |
| Specific cancer site |  |  |  |  |  |  |  |
| Digestive (reference) |  |  |  |  |  |  |  |
| Breast | 2.16 (0.86-5.44) | 0.28 (0.03-2.71) | 1.40 (0.67-2.93) | 5.16 (0.28-95.80) | 2.08 (0.50-8.62) | 0.75 (0.35-1.59) |  |
| Respiratory | 0.98 (0.48-2.02) | 0.00 (0.00-Inf) | 1.44 (0.77-2.68) | 3.83 (0.41-36.10) | 2.36 (0.92-6.06) | 1.03 (0.58-1.82) |  |
| Urologic | 0.94 (0.33-2.67) | 0.96 (0.18-5.14) | 0.67 (0.27-1.66) | 2.15 (0.15-30.50) | 0.57 (0.07-4.77) | 1.50 (0.73-3.10) |  |
| Gynecologic | 1.89 (0.56-6.40) | 0.00 (0.00-Inf) | 0.80 (0.26-2.46) | 10.20 (0.30-346.00) | 0.00 (0.00-Inf) | 0.54 (0.16-1.79) |  |
| Other | 0.94 (0.38-2.33) | 1.95 (0.60-6.32) | 1.05 (0.52-2.13) | 0.00 (0.00-Inf) | 0.92 (0.18-4.66) | 0.99 (0.51-1.92) |  |
| Never diagnosed with cancer | 4.04 (0.99-16.40) | 0.00 (0.00-Inf) | 1.17 (0.45-3.05) | 0.00 (0.00-Inf) | 0.00 (0.00-Inf) | 0.54 (0.20-1.44) |  |
| Relationship with survivor |  |  |  |  |  |  |  |
| Spouse (reference) |  |  |  |  |  |  |  |
| Child | 0.32 (0.13-0.77)* | 1.77 (0.34-9.35) | 1.34 (0.66-2.75) | 0.00 (0.00-Inf) | 2.47 (0.60-10.20) | 1.36 (0.70-2.66) |  |
| Parent | 0.20 (0.02-1.96) | 1.65 (0.26-10.30) | 0.70 (0.24-2.09) | 0.00 (0.00-Inf) | 2.83 (0.19-41.90) | 0.54 (0.17-1.67) |  |
| Sibling | 0.31 (0.09-1.03) | 0.46 (0.05-4.29) | 1.11 (0.45-2.74) | 0.66 (0.06-7.81) | 0.00 (0.00-Inf) | 0.92 (0.40-2.13) |  |
| Other | 0.31 (0.10-0.98)* | 0.47 (0.05-4.37) | 1.19 (0.53-2.69) | 0.66 (0.04-11.10) | 1.70 (0.28-10.50) | 0.84 (0.38-1.86) |  |
| Treatment course |  |  |  |  |  |  |  |
| Pretreatment (reference) |  |  |  |  |  |  |  |
| Ongoing | 4.82 (2.14-10.80)* | 1.78 (0.58-5.46) | 1.22 (0.74-2.02) | 1.92 (0.18-20.20) | 1.33 (0.43-4.12) | 0.74 (0.46-1.19) |  |
| Completed | 4.84 (2.00-11.70)* | 0.75 (0.13-4.54) | 0.69 (0.36-1.32) | 0.76 (0.04-15.60) | 2.48 (0.77-7.98) | 0.71 (0.40-1.26) |  |
| Residence |  |  |  |  |  |  |  |
| CDO† (reference) |  |  |  |  |  |  |  |
| Within KP‡ | 1.07 (0.61-1.87) | 1.49 (0.53-4.22) | 1.03 (0.64-1.66) | 1.91 (0.32-11.60) | 1.32 (0.53-3.27) | 1.05 (0.67-1.65) |  |
| Outside KP‡ | 0.54 (0.22-1.33) | 0.61 (0.07-5.14) | 1.34 (0.72-2.48) | 0.00 (0.00-Inf) | 1.08 (0.31-3.73) | 1.50 (0.84-2.68) |  |
| Symptom |  |  |  |  |  |  |  |
| Yes (reference) |  |  |  |  |  |  |  |
| No | 9.34 (3.52-24.80)* | 0.65 (0.23-1.87) | 0.68 (0.42-1.10) | 0.87 (0.14-5.54) | 1.76 (0.56-5.50) | 0.86 (0.55-1.36) |  |
| Past consultation history at KCC§ |  |  |  |  |  |  |  |
| Yes (reference) |  |  |  |  |  |  |  |
| No | 3.12 (1.00-9.71) | 2.49 (0.45-13.90) | 1.27 (0.45-3.55) | 0.00 (0.00-Inf) | 0.00 (0.00-Inf) | 0.21 (0.05-0.95)* |  |
| **p* < 0.05, †A city designated by official ordinance, ‡Kanagawa prefecture, §Kanagawa Cancer Center | | | | | | | |

| **Table A3. Logistic regression analysis (except survivor, continued)** | | | | | | |
| --- | --- | --- | --- | --- | --- | --- |
|  | Odds ratio (95% Confidence interval) | | | | | |
|  | Emotions  /Mental Health | Social Support | Communications | Provider Relationship | Cure | Employment |
| Caller's sex |  |  |  |  |  |  |
| Male(reference) |  |  |  |  |  |  |
| Female | 1.54 (0.86-2.76) | 3.34 (0.31-36.10) | 1.32 (0.66-2.65) | 0.58 (0.29-1.15) | 0.73 (0.46-1.16) | 1.05 (0.02-47.00) |
| Caller's age group (in years) |  |  |  |  |  |  |
| < 40, 40-59, ≥ 60 | 0.81 (0.50-1.32) | 0.31 (0.06-1.59) | 0.72 (0.42-1.23) | 0.65 (0.33-1.27) | 0.63 (0.42-0.96)* | 0.01 (0.00-2.36) |
| Survivor's age group (in years) |  |  |  |  |  |  |
| < 40, 40-59, 60-69, ≥ 70 | 0.76 (0.52-1.10) | 1.97 (0.59-6.57) | 0.93 (0.62-1.41) | 1.36 (0.80-2.33) | 1.21 (0.87-1.68) | 8.09 (0.19-341.00) |
| Specific cancer site |  |  |  |  |  |  |
| Digestive (reference) |  |  |  |  |  |  |
| Breast | 2.10 (0.94-4.67) | 0.00 (0.00-Inf) | 0.80 (0.30-2.18) | 0.80 (0.25-2.52) | 1.21 (0.58-2.52) | 0.00 (0.00-Inf) |
| Respiratory | 1.00 (0.48-2.10) | 1.29 (0.21-7.94) | 0.54 (0.21-1.39) | 0.38 (0.11-1.35) | 0.87 (0.48-1.58) | 16.40 (0.14-1990.00) |
| Urologic | 1.34 (0.54-3.31) | 0.00 (0.00-Inf) | 1.37 (0.52-3.57) | 0.90 (0.28-2.93) | 1.77 (0.86-3.67) | 8.08 (0.06-1170.00) |
| Gynecologic | 1.33 (0.39-4.55) | 6.78 (0.45-101.00) | 0.58 (0.12-2.78) | 6.26 (1.80-21.80)* | 0.88 (0.28-2.76) | 77.80 (0.32-18700.00) |
| Other | 1.04 (0.45-2.38) | 1.08 (0.10-11.70) | 0.25 (0.07-0.83)* | 0.73 (0.24-2.21) | 0.83 (0.42-1.65) | 0.00 (0.00-Inf) |
| Never diagnosed with cancer | 5.15 (1.95-13.60)* | 0.00 (0.00-Inf) | 0.49 (0.12-2.05) | 1.18 (0.28-4.93) | 0.54 (0.20-1.45) | 0.00 (0.00-Inf) |
| Relationship with survivor |  |  |  |  |  |  |
| Spouse (reference) |  |  |  |  |  |  |
| Child | 0.54 (0.24-1.20) | 0.10 (0.01-1.44) | 1.05 (0.40-2.72) | 0.79 (0.25-2.43) | 0.87 (0.43-1.75) | 0.00 (0.00-Inf) |
| Parent | 0.55 (0.17-1.79) | 0.00 (0.00-Inf) | 4.78 (1.24-18.50)* | 1.69 (0.32-9.06) | 1.62 (0.56-4.69) | 1360.00 (0.16-116 x10^5^) |
| Sibling | 0.62 (0.24-1.60) | 0.00 (0.00-Inf) | 1.56 (0.48-5.09) | 1.52 (0.42-5.53) | 1.05 (0.44-2.48) | 5.73 (0.12-271.00) |
| Other | 0.35 (0.13-0.97)* | 0.63 (0.04-9.80) | 2.32 (0.81-6.70) | 0.47 (0.11-1.97) | 0.62 (0.26-1.45) | 0.00 (0.00-Inf) |
| Treatment course |  |  |  |  |  |  |
| Pretreatment (reference) |  |  |  |  |  |  |
| Ongoing | 1.49 (0.82-2.70) | 1.22 (0.11-13.50) | 1.46 (0.71-2.98) | 1.85 (0.84-4.06) | 1.01 (0.62-1.63) | 0.16 (0.01-3.71) |
| Completed | 1.41 (0.69-2.87) | 2.17 (0.19-24.60) | 1.60 (0.69-3.69) | 0.70 (0.24-2.07) | 0.86 (0.48-1.54) | 0.00 (0.00-Inf) |
| Residence |  |  |  |  |  |  |
| CDO† (reference) |  |  |  |  |  |  |
| Within KP‡ | 0.87 (0.50-1.49) | 0.62 (0.10-3.66) | 0.68 (0.35-1.30) | 0.46 (0.21-1.01) | 1.27 (0.81-2.01) | 1.75 (0.04-68.70) |
| Outside KP‡ | 1.24 (0.61-2.53) | 1.21 (0.12-12.30) | 0.41 (0.15-1.12) | 0.45 (0.15-1.38) | 1.73 (0.95-3.12) | 0.00 (0.00-Inf) |
| Symptom |  |  |  |  |  |  |
| Yes (reference) |  |  |  |  |  |  |
| No | 1.37 (0.77-2.42) | 2.02 (0.23-17.90) | 1.38 (0.70-2.74) | 0.90 (0.43-1.86) | 0.68 (0.43-1.08) | 4.79 (0.14-170.00) |
| Past consultation history at KCC§ |  |  |  |  |  |  |
| Yes (reference) |  |  |  |  |  |  |
| No | 0.61 (0.16-2.30) | 0.00 (0.00-Inf) | 0.76 (0.16-3.59) | 0.18 (0.02-1.75) | 0.10 (0.01-0.75)* | 0.00 (0.00-Inf) |
| **p* < 0.05, †A city designated by official ordinance, ‡Kanagawa prefecture, §Kanagawa Cancer Center | | | | | | |
