# Supplementary material for: Impact of individual background on the unmet needs of cancer survivors and caregivers – a mixed-methods analysis
Source: BMC Cancer. 2020 Mar 30;20:263. doi: 10.1186/s12885-020-06732-5 (PMC7106842; doi:10.1186/s12885-020-06732-5)
Supplement: Supplementary file 5 — Additional file 5: Table A4. Stratified logistic regression analysis 2006–2010 (survivors). [file 12885_2020_6732_MOESM5_ESM.docx]

| **Table A4. Stratified logistic regression analysis 2006-2010 (survivors)** | | | | | | | | |
| --- | --- | --- | --- | --- | --- | --- | --- | --- |
|  | Odds ratio (95% Confidence interval) | | | | | | |  |
|  | Physical | Financial | Education/information | Personal Control | System of Care | Resources |  |  |
| Sex |  |  |  |  |  |  |  |  |
| Male (reference) |  |  |  |  |  |  |  |  |
| Female | 2.06 (1.13-3.76)* | 0.40 (0.15-1.12) | 1.05 (0.66-1.68) | 1.82 (0.45-7.34) | 1.61 (0.52-5.02) | 0.65 (0.41-1.02) |  |  |
| Age group (in years) |  |  |  |  |  |  |  |  |
| < 40, 40-59, 60-69, ≥ 70 | 1.17 (0.89-1.54) | 0.96 (0.61-1.50) | 0.93 (0.74-1.16) | 0.68 (0.35-1.32) | 0.98 (0.58-1.68) | 1.14 (0.91-1.42) |  |  |
| Cancer type |  |  |  |  |  |  |  |  |
| Breast (reference) |  |  |  |  |  |  |  |  |
| Colon | 0.98 (0.40-2.41) | 0.41 (0.09-1.80) | 1.55 (0.72-3.31) | 0.00 (0.00-Inf) | 1.79 (0.30-10.80) | 1.14 (0.50-2.60) |  |  |
| Lung | 0.55 (0.20-1.54) | 0.18 (0.02-1.68) | 1.67 (0.71-3.97) | 0.00 (0.00-Inf) | 0.00 (0.00-Inf) | 1.33 (0.55-3.25) |  |  |
| Stomach | 1.44 (0.53-3.93) | 0.12 (0.01-1.20) | 1.18 (0.47-2.95) | 2.56 (0.40-16.30) | 2.26 (0.34-15.00) | 1.65 (0.69-3.97) |  |  |
| Other | 0.69 (0.35-1.33) | 0.29 (0.09-0.93)* | 1.04 (0.58-1.88) | 1.03 (0.26-4.01) | 1.41 (0.35-5.69) | 1.27 (0.69-2.34) |  |  |
| Multi primary | 2.24 (0.72-6.96) | 0.32 (0.04-2.78) | 0.41 (0.11-1.51) | 4.89 (0.76-31.30) | 1.68 (0.17-16.90) | 1.03 (0.35-3.03) |  |  |
| Never diagnosed with cancer | 0.35 (0.13-0.95)* | 0.18 (0.04-0.79)* | 1.74 (0.89-3.41) | 0.00 (0.00-Inf) | 1.82 (0.38-8.62) | 1.23 (0.61-2.47) |  |  |
| Treatment course |  |  |  |  |  |  |  |  |
| Pretreatment (reference) |  |  |  |  |  |  |  |  |
| Ongoing | 4.72 (2.14-10.40)* | 0.56 (0.22-1.44) | 1.03 (0.63-1.68) | 0.81 (0.26-2.55) | 0.79 (0.25-2.51) | 0.46 (0.28-0.76)* |  |  |
| Completed | 4.84 (2.12-11.10)* | 0.95 (0.37-2.46) | 0.62 (0.37-1.03) | 0.00 (0.00-Inf) | 0.55 (0.16-1.86) | 0.68 (0.42-1.11) |  |  |
| Residence |  |  |  |  |  |  |  |  |
| CDO† (reference) |  |  |  |  |  |  |  |  |
| Within KP‡ | 0.70 (0.42-1.19) | 0.73 (0.31-1.73) | 0.89 (0.58-1.37) | 1.00 (0.31-3.24) | 1.32 (0.50-3.51) | 1.18 (0.78-1.78) |  |  |
| Outside KP‡ | 0.29 (0.08-1.14) | 1.03 (0.22-4.83) | 1.09 (0.50-2.38) | 1.91 (0.34-10.90) | 0.94 (0.11-7.98) | 1.27 (0.57-2.83) |  |  |
| Symptom |  |  |  |  |  |  |  |  |
| Yes (reference) |  |  |  |  |  |  |  |  |
| No | 9.36 (5.29-16.60)* | 0.52 (0.24-1.13) | 0.45 (0.30-0.66)* | 1.23 (0.40-3.73) | 1.28 (0.50-3.24) | 0.81 (0.55-1.19) |  |  |
| Past consultation history at KCC§ |  |  |  |  |  |  |  |  |
| Yes (reference) |  |  |  |  |  |  |  |  |
| No | 1.36 (0.65-2.85) | 1.22 (0.34-4.37) | 1.33 (0.68-2.61) | 0.68 (0.08-5.70) | 0.54 (0.07-4.22) | 0.35 (0.14-0.84) |  |  |
| *p < 0.05, †A city designated by official ordinance,‡Kanagawa prefecture, §Kanagawa Cancer Center | | | | | | | | |

| **Table A4. Stratified logistic regression analysis 2006-2010 (survivors, continued)** | | | | | | |
| --- | --- | --- | --- | --- | --- | --- |
|  | Odds ratio (95% Confidence interval) | | | | | |
|  | Emotions /Mental Health | Social Support | Communications | Provider Relationship | Cure | Employment |
| Sex |  |  |  |  |  |  |
| Male (reference) |  |  |  |  |  |  |
| Female | 1.72 (1.05-2.81)* | 3.15 (0.30-33.00) | 1.28 (0.51-3.26) | 1.56 (0.81-3.01) | 0.65 (0.41-1.03) | 0.20 (0.04-0.94)* |
| Age group (in years) |  |  |  |  |  |  |
| < 40, 40-59, 60-69, ≥ 70 | 0.87 (0.69-1.09) | 1.47 (0.70-3.12) | 1.24 (0.82-1.90) | 0.97 (0.71-1.32) | 1.05 (0.84-1.32) | 0.40 (0.21-0.79)* |
| Cancer type |  |  |  |  |  |  |
| Breast (reference) |  |  |  |  |  |  |
| Colon | 0.80 (0.35-1.80) | 0.00 (0.00-Inf) | 1.78 (0.56-5.65) | 1.30 (0.45-3.75) | 1.03 (0.47-2.27) | 0.00 (0.00-Inf) |
| Lung | 0.97 (0.40-2.34) | 0.00 (0.00-Inf) | 0.24 (0.03-2.07) | 2.14 (0.74-6.20) | 1.06 (0.44-2.55) | 0.36 (0.03-4.87) |
| Stomach | 0.91 (0.37-2.25) | 0.00 (0.00-Inf) | 0.29 (0.03-2.54) | 1.40 (0.43-4.58) | 0.71 (0.28-1.81) | 0.22 (0.02-2.88) |
| Other | 0.92 (0.52-1.62) | 0.21 (0.03-1.28) | 0.56 (0.20-1.59) | 1.13 (0.51-2.52) | 0.85 (0.47-1.56) | 0.49 (0.10-2.34) |
| Multi primary | 0.84 (0.29-2.41) | 1.60 (0.23-11.10) | 2.05 (0.54-7.78) | 1.82 (0.51-6.47) | 0.61 (0.20-1.89) | 0.00 (0.00-Inf) |
| Never diagnosed with cancer | 1.05 (0.53-2.07) | 0.22 (0.02-2.18) | 0.34 (0.07-1.73) | 1.19 (0.44-3.23) | 0.80 (0.39-1.64) | 0.00 (0.00-Inf) |
| Treatment course |  |  |  |  |  |  |
| Pretreatment (reference) |  |  |  |  |  |  |
| Ongoing | 1.14 (0.68-1.93) | 0.83 (0.18-3.73) | 1.16 (0.45-3.01) | 1.75 (0.82-3.72) | 0.94 (0.57-1.54) | 0.75 (0.18-3.08) |
| Completed | 1.37 (0.81-2.31) | 0.76 (0.13-4.33) | 0.66 (0.22-2.01) | 1.40 (0.64-3.07) | 0.60 (0.36-1.02) | 2.41 (0.63-9.17) |
| Residence |  |  |  |  |  |  |
| CDO† (reference) |  |  |  |  |  |  |
| Within KP‡ | 1.48 (0.97-2.25) | 0.42 (0.09-2.01) | 1.46 (0.69-3.11) | 1.34 (0.76-2.38) | 1.32 (0.87-2.01) | 1.06 (0.36-3.15) |
| Outside KP‡ | 0.64 (0.25-1.63) | 2.07 (0.21-20.50) | 0.61 (0.07-4.93) | 0.79 (0.22-2.83) | 1.94 (0.89-4.23) | 0.00 (0.00-Inf) |
| Symptom |  |  |  |  |  |  |
| Yes (reference) |  |  |  |  |  |  |
| No | 1.92 (1.28-2.86)* | 2.32 (0.64-8.47) | 2.34 (1.09-5.00)* | 1.61 (0.92-2.81) | 0.58 (0.39-0.86)* | 2.30 (0.74-7.18) |
| Past consultation history at KCC§ |  |  |  |  |  |  |
| Yes (reference) |  |  |  |  |  |  |
| No | 0.86 (0.44-1.69) | 0.00 (0.00-Inf) | 1.71 (0.60-4.86) | 0.41 (0.12-1.39) | 0.24 (0.08-0.69)* | 0.68 (0.08-5.86) |
| *p < 0.05, †A city designated by official ordinance, ‡Kanagawa prefecture, §Kanagawa Cancer Center | | | | | | |
