# Supplementary material for: Impact of individual background on the unmet needs of cancer survivors and caregivers – a mixed-methods analysis
Source: BMC Cancer. 2020 Mar 30;20:263. doi: 10.1186/s12885-020-06732-5 (PMC7106842; doi:10.1186/s12885-020-06732-5)
Supplement: Supplementary file 6 — Additional file 6: Table A5. Stratified logistic regression analysis 2011–2014 (survivors). [file 12885_2020_6732_MOESM6_ESM.docx]

| **Table A5. Stratified logistic regression analysis 2011-2014 (survivors)** | | | | | | | | |
| --- | --- | --- | --- | --- | --- | --- | --- | --- |
|  | Odds ratio (95% Confidence interval) | | | | | | |  |
|  | Physical | Financial | Education/information | Personal Control | System of Care | Resources |  |  |
| Sex |  |  |  |  |  |  |  |  |
| Male (reference) |  |  |  |  |  |  |  |  |
| Female | 4.42 (1.70-11.50)* | 0.59 (0.12-3.00) | 0.39 (0.19-0.80)* | 3.25 (0.24-44.70) | 0.53 (0.07-4.08) | 0.50 (0.23-1.11) |  |  |
| Age group (in years) |  |  |  |  |  |  |  |  |
| < 40, 40-59, 60-69, ≥ 70 | 1.94 (1.21-3.12)* | 1.13 (0.50-2.57) | 0.91 (0.63-1.31) | 3.33 (0.64-17.30) | 0.42 (0.13-1.34) | 0.63 (0.42-0.95)* |  |  |
| Cancer type |  |  |  |  |  |  |  |  |
| Breast (reference) |  |  |  |  |  |  |  |  |
| Colon | 1.18 (0.37-3.82) | 0.81 (0.07-10.10) | 0.48 (0.14-1.62) | 1.66 (0.07-37.20) | 2.62 (0.11-60.40) | 2.04 (0.64-6.48) |  |  |
| Lung | 1.48 (0.30-7.30) | 0.00 (0.00-Inf) | 1.56 (0.47-5.25) | 4.17 (0.16-110.00) | 1.65 (0.06-48.10) | 1.10 (0.28-4.26) |  |  |
| Stomach | 0.35 (0.08-1.48) | 0.91 (0.07-11.70) | 1.34 (0.42-4.26) | 0.00 (0.00-Inf) | 0.00 (0.00-Inf) | 1.14 (0.29-4.46) |  |  |
| Other | 0.62 (0.21-1.83) | 1.74 (0.26-11.90) | 1.14 (0.45-2.88) | 0.71 (0.02-21.10) | 1.29 (0.10-16.80) | 0.88 (0.32-2.46) |  |  |
| Multi primary | 2.40 (0.13-46.10) | 0.00 (0.00-Inf) | 0.62 (0.05-7.22) | 0.00 (0.00-Inf) | 0.00 (0.00-Inf) | 1.33 (0.10-17.10) |  |  |
| Never diagnosed with cancer | 0.45 (0.07-2.98) | 0.00 (0.00-Inf) | 1.32 (0.40-4.41) | 0.00 (0.00-Inf) | 1.44 (0.07-28.10) | 1.41 (0.41-4.87) |  |  |
| Treatment course |  |  |  |  |  |  |  |  |
| Pretreatment (reference) |  |  |  |  |  |  |  |  |
| Ongoing | 7.22 (2.01-25.90)* | 0.91 (0.17-4.94) | 1.70 (0.74-3.91) | 159 x 10^8^ (0.00-Inf) | 0.00 (0.00-Inf) | 0.39 (0.15-1.04) |  |  |
| Completed | 7.55 (2.19-26.00)* | 0.75 (0.14-4.08) | 1.22 (0.57-2.57) | 1.22 x 10^8^ (0.00-Inf) | 1.00 (0.15-6.56) | 0.52 (0.24-1.15) |  |  |
| Residence |  |  |  |  |  |  |  |  |
| CDO† (reference) |  |  |  |  |  |  |  |  |
| Within KP‡ | 3.52 (1.56-7.94)* | 1.35 (0.32-5.64) | 0.51 (0.26-1.02) | 4.42 (0.32-61.00) | 1.49 (0.23-9.64) | 1.09 (0.53-2.27) |  |  |
| Outside KP‡ | 0.00 (0.00-Inf) | 0.00 (0.00-Inf) | 0.44 (0.09-2.24) | 0.00 (0.00-Inf) | 0.00 (0.00-Inf) | 2.88 (0.75-11.10) |  |  |
| Symptom |  |  |  |  |  |  |  |  |
| Yes (reference) |  |  |  |  |  |  |  |  |
| No | 8.86 (3.22-24.40)* | 1.67 (0.40-7.08) | 0.50 (0.26-0.96)* | 0.43 (0.05-4.13) | 0.26 (0.03-2.43) | 0.55 (0.27-1.10) |  |  |
| Past consultation history at KCC§ |  |  |  |  |  |  |  |  |
| Yes (reference) |  |  |  |  |  |  |  |  |
| No | 0.87 (0.23-3.24) | 6.34 (1.35-29.80) | 0.81 (0.28-2.36) | 0.00 (0.00-Inf) | 2.50 (0.17-37.80) | 1.38 (0.44-4.34) |  |  |
| *p < 0.05, †A city designated by official ordinance,‡Kanagawa prefecture, §Kanagawa Cancer Center | | | | | | | | |

| **Table A5. Stratified logistic regression analysis 2011-2014 (survivors, continued)** | | | | | | |
| --- | --- | --- | --- | --- | --- | --- |
|  | Odds ratio (95% Confidence interval) | | | | | |
|  | Emotions /Mental Health | Social Support | Communications | Provider Relationship | Cure | Employment |
| Sex |  |  |  |  |  |  |
| Male (reference) |  |  |  |  |  |  |
| Female | 1.21 (0.47-3.11) | 1.23 (0.07-21.00) | 9.90 (0.88-112.00) | 0.58 (0.18-1.86) | 0.99 (0.40-2.43) | 3.86 x 10^7^ (0.00-Inf) |
| Age group (in years) |  |  |  |  |  |  |
| < 40, 40-59, 60-69, ≥ 70 | 1.32 (0.83-2.10) | 0.65 (0.11-3.63) | 1.26 (0.58-2.76) | 0.99 (0.54-1.83) | 0.89 (0.57-1.41) | 0.28 (0.06-1.35) |
| Cancer type |  |  |  |  |  |  |
| Breast (reference) |  |  |  |  |  |  |
| Colon | 0.76 (0.22-2.65) | 0.44 (0.00-Inf) | 0.72 (0.06-8.35) | 3.31 (0.73-15.00) | 0.92 (0.16-5.12) | 0.00 (0.00-Inf) |
| Lung | 0.00 (0.00-Inf) | 1.64 (0.00-Inf) | 1.03 (0.09-12.20) | 0.64 (0.06-7.27) | 2.94 (0.71-12.20) | 0.00 (0.00-Inf) |
| Stomach | 0.68 (0.17-2.67) | 1.14 x 10^17^ (0.00-Inf) | 1.12 (0.10-12.20) | 0.54 (0.05-5.91) | 2.66 (0.64-11.00) | 0.00 (0.00-Inf) |
| Other | 0.40 (0.13-1.27) | 8.73 x 10^8^ (0.00-Inf) | 0.63 (0.09-4.30) | 0.91 (0.18-4.54) | 1.71 (0.55-5.35) | 0.87 (0.11-7.07) |
| Multi primary | 0.00 (0.00-Inf) | 1.48 x 10^8^ (0.00-Inf) | 0.00 (0.00-Inf) | 0.00 (0.00-Inf) | 4.57 (0.35-59.00) | 0.00 (0.00-Inf) |
| Never diagnosed with cancer | 2.21 (0.64-7.59) | 0.95 (0.00-Inf) | 0.00 (0.00-Inf) | 1.33 (0.18-9.78) | 0.62 (0.11-3.63) | 0.00 (0.00-Inf) |
| Treatment course |  |  |  |  |  |  |
| Pretreatment (reference) |  |  |  |  |  |  |
| Ongoing | 0.49 (0.16-1.46) | 4.58 x 10^16^ (0.00-Inf) | 5.74 (0.97-34.00) | 0.85 (0.21-3.44) | 0.39 (0.14-1.09) | 5.32 (0.23-122.00) |
| Completed | 0.86 (0.34-2.17) | 6.55 x 10^16^ (0.00-Inf) | 0.97 (0.12-7.69) | 0.98 (0.29-3.36) | 0.23 (0.08-0.65)* | 8.80 (0.63-122.00) |
| Residence |  |  |  |  |  |  |
| CDO† (reference) |  |  |  |  |  |  |
| Within KP‡ | 0.72 (0.30-1.77) | 0.00 (0.00-Inf) | 0.67 (0.15-3.10) | 0.88 (0.28-2.84) | 0.90 (0.38-2.10) | 1.12 (0.17-7.37) |
| Outside KP‡ | 1.92 (0.34-11.00) | 0.14 (0.00-Inf) | 0.00 (0.00-Inf) | 1.17 (0.12-11.80) | 1.63 (0.35-7.69) | 0.00 (0.00-Inf) |
| Symptom |  |  |  |  |  |  |
| Yes (reference) |  |  |  |  |  |  |
| No | 2.08 (0.90-4.85) | 0.00 (0.00-Inf) | 0.16 (0.04-0.72)* | 1.45 (0.50-4.20) | 0.85 (0.37-1.95) | 0.41 (0.06-2.75) |
| Past consultation history at KCC§ |  |  |  |  |  |  |
| Yes (reference) |  |  |  |  |  |  |
| No | 0.56 (0.12-2.67) | 0.00 (0.00-Inf) | 0.00 (0.00-Inf) | 1.64 (0.33-8.16) | 0.70 (0.15-3.38) | 0.00 (0.00-Inf) |
| *p < 0.05, †A city designated by official ordinance, ‡Kanagawa prefecture, §Kanagawa Cancer Center | | | | | | |
