# Supplementary material for: Impact of individual background on the unmet needs of cancer survivors and caregivers – a mixed-methods analysis
Source: BMC Cancer. 2020 Mar 30;20:263. doi: 10.1186/s12885-020-06732-5 (PMC7106842; doi:10.1186/s12885-020-06732-5)
Supplement: Supplementary file 7 — Additional file 7: Table A6. Logistic regression analysis (including both survivors and caregivers). [file 12885_2020_6732_MOESM7_ESM.docx]

| **Table A6. Logistic regression analysis (including both survivors and caregivers)** | | | | | | |
| --- | --- | --- | --- | --- | --- | --- |
|  | Odds ratio (95% Confidence interval) | | | | | |
|  | Physical | Financial | Education/Information | Personal Control | System of Care | Resources |
| Caller's sex |  |  |  |  |  |  |
| Male (reference) |  |  |  |  |  |  |
| Female | 2.06 (1.48-2.87)* | 0.93 (0.53-1.60) | 0.78 (0.61-1.01) | 1.71 (0.66-4.47) | 1.21 (0.69-2.15)* | 0.74 (0.58-0.94)* |
| Caller's age group (in years) |  |  |  |  |  |  |
| < 40, 40-59, ≥ 60 | 1.25 (1.01-1.54)* | 1.09 (0.75-1.59) | 0.97 (0.82-1.15) | 0.91 (0.51-1.64) | 0.92 (0.65-1.32) | 0.93 (0.79-1.09) |
| Cancer type |  |  |  |  |  |  |
| Breast (reference) |  |  |  |  |  |  |
| Colon | 0.76 (0.45-1.27) | 1.15 (0.50-2.65) | 0.88 (0.56-1.38) | 0.64 (0.13-3.21) | 1.01 (0.38-2.71) | 1.54 (0.97-2.43) |
| Lung | 0.76 (0.45-1.30) | 0.11 (0.01-0.83)* | 1.49 (0.95-2.33) | 1.13 (0.27-4.80) | 1.49 (0.60-3.73) | 1.66 (1.05-2.63)* |
| Stomach | 0.89 (0.51-1.55) | 0.61 (0.21-1.80) | 1.14 (0.71-1.83) | 0.89 (0.17-4.70) | 0.53 (0.16-1.80) | 1.44 (0.89-2.32) |
| Other | 0.76 (0.50-1.14) | 0.97 (0.49-1.92) | 0.90 (0.63-1.28) | 1.08 (0.38-3.09) | 0.86 (0.38-1.92) | 1.62 (1.12-2.34)* |
| Multi primary | 1.31 (0.64-2.68) | 0.31 (0.04-2.44) | 0.47 (0.21-1.07) | 2.06 (0.39-10.80) | 1.56 (0.46-5.35) | 1.55 (0.80-3.03) |
| Never diagnosed with cancer | 0.51 (0.26-1.02) | 0.31 (0.08-1.12) | 1.43 (0.90-2.27) | 0.00 (0.00-Inf) | 0.74 (0.24-2.31) | 1.28 (0.79-2.08) |
| Relationship with survivor |  |  |  |  |  |  |
| Survivor (reference) |  |  |  |  |  |  |
| Caregiver | 0.61 (0.45-0.83)* | 0.90 (0.52-1.55) | 0.97 (0.76-1.26) | 0.34 (0.13-0.85)* | 1.56 (0.88-2.74) | 1.56 (1.22-1.99)* |
| Treatment course |  |  |  |  |  |  |
| Pretreatment (reference) |  |  |  |  |  |  |
| Ongoing | 4.38 (2.81-6.84)* | 0.98 (0.54-1.79) | 1.30 (0.98-1.72) | 1.66 (0.65-4.22) | 0.78 (0.41-1.49) | 0.60 (0.46-0.79)* |
| Completed | 4.77 (3.01-7.57)* | 0.81 (0.41-1.60) | 0.83 (0.61-1.13) | 0.40 (0.10-1.62) | 1.43 (0.76-2.67) | 0.70 (0.53-0.93)* |
| Residence |  |  |  |  |  |  |
| CDO† (reference) |  |  |  |  |  |  |
| Within KP‡ | 0.99 (0.73-1.34) | 0.97 (0.57-1.65) | 0.92 (0.71-1.18) | 1.31 (0.57-2.98) | 1.29 (0.76-2.20) | 1.08 (0.85-1.37) |
| Outside KP‡ | 0.55 (0.32-0.96)* | 0.46 (0.14-1.51) | 1.43 (0.98-2.10) | 1.42 (0.39-5.13) | 0.92 (0.38-2.25) | 1.19 (0.82-1.73) |
| Symptom |  |  |  |  |  |  |
| Yes (reference) |  |  |  |  |  |  |
| No | 8.60 (5.73-12.90)* | 0.65 (0.39-1.07) | 0.57 (0.45-0.73)* | 0.95 (0.42-2.14) | 1.24 (0.71-2.15) | 0.74 (0.59-0.94)* |
| Past consultation history at KCC§ |  |  |  |  |  |  |
| Yes (reference) |  |  |  |  |  |  |
| No | 1.61 (0.99-2.60) | 1.91 (0.91-4.01) | 1.00 (0.64-1.56) | 0.43 (0.06-3.23) | 0.60 (0.19-1.97) | 0.37 (0.21-0.64)* |
| *p < 0.05, †A city designated by official ordinance, ‡Kanagawa prefecture, §Kanagawa Cancer Center | | | | | | |

| **Table A6. Logistic regression analysis (including both survivors and caregivers, continued)** | | | | | | |
| --- | --- | --- | --- | --- | --- | --- |
|  | Odds ratio (95% Confidence interval) | | | | | |
|  | Emotions  /Mental Health | Social Support | Communications | Provider Relationship | Cure | Employment |
| Caller's sex |  |  |  |  |  |  |
| Male (reference) |  |  |  |  |  |  |
| Female | 1.50 (1.12-2.02)* | 2.71 (0.89-8.28) | 1.62 (1.05-2.51)* | 0.83 (0.58-1.20) | 0.78 (0.61-1.01) | 0.59 (0.23-1.52) |
| Caller's age group (in years) |  |  |  |  |  |  |
| < 40, 40-59, ≥ 60 | 1.00 (0.83-1.20) | 0.98 (0.55-1.75) | 0.98 (0.75-1.27) | 0.82 (0.64-1.05) | 0.88 (0.74-1.04) | 0.46 (0.26-0.83)* |
| Cancer type |  |  |  |  |  |  |
| Breast (reference) |  |  |  |  |  |  |
| Colon | 0.54 (0.32-0.91)* | 0.44 (0.09-2.25) | 0.87 (0.45-1.69) | 1.09 (0.56-2.13) | 1.00 (0.63-1.59) | 0.00 (0.00-Inf) |
| Lung | 0.64 (0.38-1.07) | 0.49 (0.09-2.60) | 0.50 (0.23-1.08) | 1.32 (0.67-2.60) | 1.15 (0.72-1.83) | 0.51 (0.10-2.70) |
| Stomach | 0.80 (0.47-1.34) | 0.98 (0.23-4.15) | 0.83 (0.40-1.74) | 1.20 (0.59-2.45) | 0.90 (0.55-1.48) | 0.43 (0.08-2.37) |
| Other | 0.76 (0.52-1.10) | 0.81 (0.28-2.32) | 0.80 (0.47-1.37) | 1.23 (0.72-2.09) | 1.19 (0.83-1.72) | 0.78 (0.28-2.12) |
| Multi primary | 0.58 (0.27-1.26) | 1.41 (0.27-7.33) | 1.38 (0.57-3.35) | 1.57 (0.63-3.92) | 1.06 (0.53-2.13) | 0.00 (0.00-Inf) |
| Never diagnosed with cancer | 1.66 (1.03-2.66)* | 0.33 (0.04-2.84) | 0.49 (0.19-1.27) | 1.23 (0.58-2.59) | 0.81 (0.49-1.36) | 0.00 (0.00-Inf) |
| Relationship with survivor |  |  |  |  |  |  |
| Survivor (reference) |  |  |  |  |  |  |
| Caregiver | 0.77 (0.58-1.02) | 0.80 (0.33-1.93) | 2.13 (1.40-3.24)* | 0.84 (0.57-1.21) | 1.39 (1.07-1.79) | 0.20 (0.07-0.57) |
| Treatment course |  |  |  |  |  |  |
| Pretreatment (reference) |  |  |  |  |  |  |
| Ongoing | 1.27 (0.92-1.76) | 1.92 (0.60-6.15) | 1.55 (0.97-2.48) | 1.75 (1.13-2.71)* | 1.01 (0.76-1.34) | 1.01 (0.36-2.85) |
| Completed | 1.29 (0.92-1.80) | 2.24 (0.67-7.42) | 1.04 (0.61-1.79) | 1.20 (0.74-1.94) | 0.63 (0.46-0.87)* | 1.71 (0.62-4.73) |
| Residence |  |  |  |  |  |  |
| CDO† (reference) |  |  |  |  |  |  |
| Within KP‡ | 1.07 (0.81-1.41) | 0.49 (0.18-1.31) | 0.95 (0.64-1.42) | 0.76 (0.52-1.11) | 1.20 (0.93-1.54) | 1.20 (0.54-2.67) |
| Outside KP‡ | 0.91 (0.57-1.45) | 0.64 (0.14-2.83) | 0.49 (0.23-1.05) | 0.61 (0.31-1.17) | 1.59 (1.08-2.32)* | 0.00 (0.00-Inf) |
| Symptom |  |  |  |  |  |  |
| Yes (reference) |  |  |  |  |  |  |
| No | 1.39 (1.06-1.82)* | 1.53 (0.62-3.74) | 1.23 (0.82-1.85) | 1.28 (0.89-1.85) | 0.70 (0.54-0.89)* | 1.22 (0.53-2.83) |
| Past consultation history at KCC§ |  |  |  |  |  |  |
| Yes (reference) |  |  |  |  |  |  |
| No | 0.97 (0.61-1.56) | 0.78 (0.18-3.40) | 1.06 (0.53-2.12) | 0.47 (0.20-1.10) | 0.23 (0.12-0.47)* | 0.83 (0.19-3.66) |
| *p < 0.05, †A city designated by official ordinance, ‡Kanagawa prefecture, §Kanagawa Cancer Center | | | | | | |
